# Directed-Mutagenesis of *Flavobacterium meningosepticum* Prolyl-Oligopeptidase and a Glutamine-Specific Endopeptidase From Barley

**DOI:** 10.3389/fnut.2020.00011

**Published:** 2020-02-18

**Authors:** Claudia E. Osorio, Nuan Wen, Jaime H. Mejías, Shannon Mitchell, Diter von Wettstein, Sachin Rustgi

**Affiliations:** ^1^Department of Crop and Soil Sciences, Washington State University, Pullman, WA, United States; ^2^Agriaquaculture Nutritional Genomic Center, Temuco, Chile; ^3^Centro Regional de Investigación Carillanca, Instituto de Investigaciones Agropecuarias INIA, Temuco, Chile; ^4^Department of Biological Systems Engineering, Washington State University, Pullman, WA, United States; ^5^Department of Plant and Environmental Sciences, Clemson University Pee Dee Research and Education Center, Florence, SC, United States

**Keywords:** glutenases, site-directed mutagenesis, thermostability, glutenin, gliadin, celiac disease

## Abstract

Wheat gluten proteins are the known cause of celiac disease. The repetitive tracts of proline and glutamine residues in these proteins make them exceptionally resilient to digestion in the gastrointestinal tract. These indigested peptides trigger immune reactions in susceptible individuals, which could be either an allergic reaction or celiac disease. Gluten exclusion diet is the only approved remedy for such disorders. Recently, a combination of a glutamine specific endoprotease from barley (EP-B2), and a prolyl endopeptidase from *Flavobacterium meningosepticum* (Fm-PEP), when expressed in the wheat endosperm, were shown to reasonably detoxify immunogenic gluten peptides under simulated gastrointestinal conditions. However useful, these “glutenases” are limited in application due to their denaturation at high temperatures, which most of the food processes require. Variants of these enzymes from thermophilic organisms exist, but cannot be applied directly due to their optimum activity at temperatures higher than 37°C. Though, these enzymes can serve as a reference to guide the evolution of peptidases of mesophilic origin toward thermostability. Therefore, a sequence guided site-saturation mutagenesis approach was used here to introduce mutations in the genes encoding Fm-PEP and EP-B2. A thermostable variant of Fm-PEP capable of surviving temperatures up to 90°C and EP-B2 variant with a thermostability of up 60°C were identified using this approach. However, the level of thermostability achieved is not sufficient; the present study has provided evidence that the thermostability of glutenases can be improved. And this pilot study has paved the way for more detailed structural studies in the future to obtain variants of Fm-PEP and EP-B2 that can survive temperatures ~100°C to allow their packing in grains and use of such grains in the food industry.

## Introduction

Celiac disease with an autoimmune component affects about 1.4% of the global population (1). Currently, there is only one approved therapy for celiac disease, which is the lifelong gluten abstinence (2, 3). The effects of this prescription on individuals and families, makes it difficult to follow, due to cultural, social, technical, and financial concerns (4, 5) as well as problems associated with the use of the gluten-free commodities (6, 7). Therefore, alternative therapies are continuously being sought worldwide.

Gluten, the causal agent of celiac disease (8), is a complex mixture of polypeptides, which is also responsible for the unique technological properties to wheat (6, 9–11). Glutamine (Gln or Q, 35%) and proline (Pro or P, 15%) are the two major constituents of the gluten proteins, which give it its identity as prolamins (12). The iterative tracts of glutamine and proline-residues present in gluten proteins allow dense packing of nitrogen in grains, but also render gluten proteins highly resistant to gastric and pancreatic proteases thus producing a broad size range of Pro/Gln-rich peptides (8, 13). These indigestible peptides pass through the intestinal epithelium and reach the lamina propria where they get deamidated by the tissue transglutaminase 2 (tTG2), which increases their affinity to the human leukocyte antigen (HLA)-DQ2 or HLA-DQ8 (14, 15). Deamidated gluten peptide-DQ2 complex enhances the inflammatory gut mucosa response by eliciting an increase in the CD4+ T-helper 1 (Th 1) cell-mediated inflammation, which ultimately leads to the destruction of the intestinal microvilli (16, 17).

A strict gluten-free diet ameliorates the intestinal mucosa morphology in a large number of celiac patients. However, a prolonged reliance on such a diet leads to often ignored unwanted effects, such as poor gut health due to changes in microbial population of the gut (18) or increase in the body mass index due to an excess consumption of starch laden low-fiber content processed foods (6). Therefore, alternative methods are continually being sought. Among them, the processing methods include sourdough fermentation, use of gluten sequestering polymers or resins, food-grade enzymes of *Aspergillus* spp. (aspergillopepsin and dipeptidyl peptidase), microbial transglutaminase enzyme and flour derived from germinated or UV treated grains (6). Besides these processing methods, a large number of preventive methods, and intestinal barrier enhancing or immune targeted therapies are being developed and in various phases of clinical testing [cf. (19)]. Likewise, the use of “glutenases” and reduced-gluten wheat genotypes have also been subjected to testing (20–23). Among these approaches, the utilization of glutenases presents several advantages over the reduced-gluten wheat genotypes or other processing methods. Such as, with the use of glutenases, gluten contamination (at any level from farm to fork) can be handled without any extra effort. Additionally, the glutenases could be used in two ways as a food supplement ingested with or before each meal or by ectopically expressing these enzymes in the grains of plants producing gluten proteins. Also, glutenases do not lead to the production of bitter-tasting peptides often produced during the partial hydrolysis of gluten proteins via the food-grade enzymes (24, 25).

A large number of proteases of microbial, plant and synthetic origin have been proposed to be useful in reducing the content of immunogenic gluten peptides and epitopes (26–32). Most of these enzymes, such as pseudolysin (IasB) from *Pseudomonas aeruginosa* (33), nepenthesin from pitcher plants (31, 34), endopeptidase 40 from the soil actinomycete *Actinoallomurus* A8 (32), and an unknown enzyme from human salivary plaques (35), are at early stages of testing but seems to hold great promise. And a handful of these glutenases are already under advanced clinical trials (36). Monotherapies such as *A. niger* prolyl endopeptidase (AN-PEP), and a modified recombinant *Alicyclobacillus sendaiensis* endopeptidase (KumaMax, now Kuma030) (37, 38), and combination therapy (ALV003, now latiglutenase), which is a cocktail of a modified recombinant *Sphingomonas capsulate* prolyl endopeptidase (ALV002) and a barley endoprotease (ALV001), are among them. Specifically, latiglutenase is now under phase II clinical trials (36, 39). However, none of these therapies except AN-PEP with a tradename “Tolerase® G” are commercially available (40). Among these proposed treatments, the combination therapy, which relies on the action of two specifically selected peptidases with complementing properties (e.g., target specificity, substrate length, optimal pH, and site of action), offer specific advantages, i.e., capability to detoxify different gluten proteins in the diet, before they trigger an immune response in the gut.

Following the leads from the earlier research, our *in-silico* analysis (13), and *in vitro* gut simulation studies performed by (28), we expressed a combination of peptidases, a recombinant modified *Flavobacterium meningosepticum* prolyl endopeptidase (Fm-PEP) and a glutamine-specific endoprotease from barley (EP-B2) in the wheat endosperm (23). These two enzymes complement each other in their gluten processing properties, EP-B2 is a cysteine endopeptidase, which cuts at glutamine residues and prefers intact proteins as substrate. Whereas, Fm-PEP a serine endopeptidase, presents a substrate preference of 30 amino acids, cutting after proline residues. They also present complementary action in different portions of the digestive system, EP-B2 functions optimally in acidic pH ~4 (in the stomach), and Fm-PEP prefers neutral pH (in the duodenum) (41). The initial *in vitro* studies cast some concern on pepsin sensitivity of the Fm-PEP, but later *in vivo* study in rats showed that the enzyme survives the gastric and brush border enzymes and shows up to 60% gastric activity (42).

Even though expressing glutenases in the wheat endosperm is an attractive approach, it poses a few technical challenges, such as the enzymes expressed in grains have to go through the harsh food processing conditions, specifically high temperatures, limiting the industrial application of this approach. When tested *in vitro*, the available thermostable variant of Fm-PEP (43) showed a stark decline in the activity at temperatures above 60°C and the same happens to EP-B2 at even lower temperature (above 50°C). However, under *in vivo* conditions, the propeptide of EP-B2 serves as an intramolecular chaperone, helping the enzyme refold to its native state after thermal denaturation. Also, the signal peptide-guided sequestration of both EP-B2 and Fm-PEP into protein bodies of the endosperm cells is expected to provide thermal stability to enzymes during the baking process with minimal effect on their catalytic properties. These assumptions, however, need to be tested in a real-life scale food processing experiments, which will be undertaken on the availability of the required amount of genetically stable seeds from the selected transformants.

An alternative to overcome this challenge and to ensure the enzyme stability under food processing conditions is to engineer enzymes for thermostability or to retain biological activity after being exposed to temperatures at or over 100°C, a temperature often used in industrial food processes. Therefore, this research was designed to set the basis for engineering thermostable variants of Fm-PEP and EP-B2 using a sequence guided mutagenesis approach, with a future objective to develop transgenic wheat lines expressing these enzymes in grains. Glutenases in such lines are expected to retain activity even after getting exposed to high temperatures and, upon consumption, the ability to detoxify immunogenic gluten peptides in the human gastrointestinal tract. The results of engineering glutenases and their biochemical characterization are presented in this manuscript.

## Materials and Methods

### Materials

The plasmid containing barley EP-B2 in pET28 background was a gift from Dr. Chaitan Khosla of Stanford University. All reagents used in this study were analytical grade, until and unless notified and were purchased from Sigma.

### Selection of Sites and Type of Changes to Induce Mutations in the Genes Encoding Fm-PEP and EP-B2

Two approaches were adopted to identify sites and types of changes to be induced in the genes encoding Fm-PEP and EP-B2. In the case of Fm-PEP, sequences of prolyl endopeptidases from thermophilic organisms were identified via BLASTP searches against NCBI non-redundant (nr) protein database, and the conserved sites in these sequences were compared with the corresponding sites in the porcine-PEP (NP_001004050.1), *Pyrococcus furiosus* (Pfu)-PEP (AAA73423.1), and Fm-PEP sequences. The clustering of sequences was performed using ClustalW (see Figure S2).

To identify sites and residues for mutagenesis in EP-B2, the sequence of a thermostable cysteine endoprotease, Ervatamin C, was used as a reference (44, 45). To identify corresponding sites the amino acid sequences of EP-B2 and Ervatamin C were aligned, and the three EP-B2 sites (Val34, Gly38, and Lys180) were marked to substitute, respectively with Ser, Ser, and Ala residues in the Ervatamin C sequence [Table 1; also see Wen, (46) for details].

**Table 1:** List of amino acid residues selected for introducing substitutions in *Hordeum vulgare* cysteine endopeptidase B2 (EP-B2) sequence.

**Organism**	**Enzyme**	***T*_max_ (°C)**	**Amino acid locations**		
*Ervatamia coronaria*	Ervatamin C	70	S_(32)_	S_(36)_	A_(172)_
*Carica papaya*	Papain	50	V_(32)_	G_(36)_	K_(174)_
*Hordeum vulgare*	EP-B2	56	V	G	K

*Amino acid locations are provided in accordance with the EP-B2 protein sequence. T_max_= Temperature of maximum enzymatic activity*.

### Introduction of Selected Mutations in Fm-PEP and EP-B2

A codon-optimized version of Fm-PEP with a GC content of 61% was synthesized from GenScript, USA, and cloned into pUC57 vector. The gene was amplified from the plasmid (pUC57+Fmen) DNA to introduce point mutations, using specific primers (F: 5′-CGCCATATGAAGTACAACAAGCT-3′ and R: 5′-CGCGAGCTCCTACTTCAAACTCT-3′) flanked on either side by the *Nde*I and *Sac*I restriction sites to facilitate cloning. The PCR conditions used to amplify the gene fragment were as follows: initial melting at 98°C for 3 min; followed by 25 cycles at 98°C for 10 s, 63.7°C for 30 s, and 72°C for 3 min, and a final extension for 10 min at 72°C. Following PCR amplification, a 5 μl aliquot from the 25 μl reaction was loaded onto the 1% (w/v) agarose gel and electrophoresed for 60 min. After testing the product on the gel, 1 μl of it was ligated into pGEM®-T Easy vector following the manufacturer's instructions and transformed in *E. coli* DH5α cells. Positive colonies were selected by blue-white screening. The plasmid was isolated from positive colonies, and the presence of the desired plasmid was confirmed by restriction digestion with *Nde*I and *Sac*I. After electrophoresis, the insert was purified from the gel using Geneclean III kit (MP Biomedicals, USA) and ligated into the pET28b(+) vector also digested with the same restriction enzymes. The resultant plasmid was used to transform BL21(DE3) cells. The positive colonies containing the plasmid pET28b(+)(FmenWT) were selected and grown on liquid LB medium supplemented with 50 μg/ml kanamycin. Plasmid DNA was isolated using NucleoSpin Plasmid—plasmid Miniprep kit (Macherey Nagel, USA) following the manufacturer's instructions.

**Figure 1:**
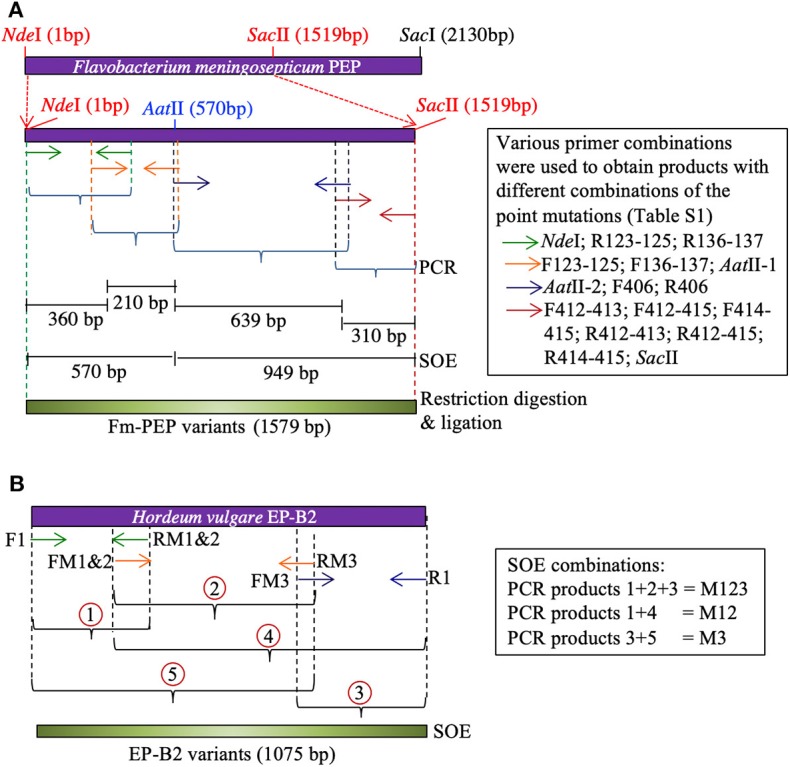
Schematic representation of Splicing by Overlapping Extension (SOE)-PCR used as a strategy to introduce mutations in the genes encoding **(A)** *Flavobacterium meningosepticum* prolyl endoprotease, and **(B)** *Hordeum vulgare* cysteine endopeptidase B2. Corresponding primers are shown as same color arrows. For primer sequences, see Table S1.

Primers were designed to introduce two mutations at a time in each blade of the Fm-PEP β-propeller domain [see above for the selection of target sites, Table S1 for primer details, and Osorio, (47) for other pertinent details]. For this purpose, the gene was divided into four sections, and unique restriction sites were used to splice together desired gene fragments. Using this approach, different mutant combinations or haplotypes were created, and the assembled gene fragments were cloned in pET28b(+) backbone using the In-Fusion HD Cloning Kit (Clontech Laboratories, Mountain View, CA) (Figure 1A).

To create the thermostable variant of EP-B2, error-prone PCR was used following Uchiyama et al. (48). The gene was divided into three sections. Overlapping primers containing desired point mutations were designed to amplify each section [see Wen, (46)]. To obtain the complete gene sequence with desired modifications, five derived PCR products were mixed in different combinations to serve as the template in splicing by overlap extension reaction (Figure 1B). The first two-point mutations were close to each other, therefore, were introduced via a single primer. The single (M3, K180A), double (M1&2, V34S, and G38S), and triple (M1&2&3, V34S, G38S, and K180A) EP-B2 mutants thus obtained, were confirmed by Sanger sequencing of the corresponding clones.

### Expression of Fm-PEP and EP-B2 Variants in *E. coli*

Colonies with the desired mutations were cultured into 5 ml LB medium with 50 μg/ml kanamycin at 37°C until OD_600_ 0.6 was reached. At that point, IPTG was added to the cultures at a final concentration of 0.25 mM and induced for another 14 h at 37°C under constant shaking (200 rpm). The cells were harvested by centrifugation at 10,000 g for 5 min, the supernatant discarded, and inclusion bodies were isolated using BugBuster™ protein extraction reagent (Novagen, USA), following the manufacturer's instructions.

The solubilization of the inclusion bodies was achieved following the protocol of Singh and Panda with minor modifications (49). Fifty microliter of inclusion body suspension at a concentration of 20 mg/ml, was solubilized in 500 μl of solubilization buffer (100 mM Tris, 2 M urea, pH 12.5). The suspension was incubated for 30 min with gentle shaking at room temperature and centrifuged at 14,000 rpm for 40 min. The supernatant was recovered, and protein concentration was measured using Bradford assay (BioRad, USA). The refolding of the extracted protein was accomplished by diluting the solubilized inclusion bodies to a final concentration of 50 μg/ml in refolding buffer pH 8.0 containing 50 mM Tris-HCl, 2 M urea, 5% sucrose, 10% glycerol, 0.5 mM EDTA, and 1 mM PMSF. Tubes were incubated overnight with gentle shaking at 4°C. The refolded sample was concentrated using Millipore concentrating devices following the manufacturer's instructions. For proEP-B2, the refolding and activation of the enzyme were accomplished as documented earlier [(41, 50); Text S1 and Figure S1].

### Evaluation of Thermostability of Fm-PEP and EP-B2 Variants

Initial screening of the mutants was performed to identify the best variants. Protein concentration was measured using the standard Bradford Protein Assay following the manufacturer's instructions (BioRad, USA). Absorbance was measured at 595 nm, and the concentration was calculated using bovine serum albumin (BSA; New England Biolabs, USA) as standard. Activity assays were performed in triplicate for each variant. Prolyl endopeptidase activity was evaluated using a synthetic peptide Z-Gly-Pro-pNA (Bachem, Torrance, CA) as described below: 100 μl aliquots of protein solution were incubated for 10 min at different temperatures starting from 60 to 90°C, with increments of 10°C. After heat treatment, the protein solution was added to 140 μl of PEP assay buffer (100 mM potassium phosphate, pH 7.0; 100 μg/ml BSA; 1 mM dithiothreitol; 0.2 mM Z-Gly-Pro-pNA to a final concentration of 0.02 μM) and incubated for 15 min at 30°C.

EP-B2 activity was analyzed using a synthetic peptide Z-Phe-Arg-pNA (Bachem, Torrance, CA). Protein concentration was measured and normalized as described above. For determination of mutant activity, a total volume of 35 μl of the proEP-B2 solution was heated for 10 min at temperatures ranging from 50 to 70°C, with increments of 2°C. Following heat-treatment, the proenzyme was activated to EP-B2, as described above. Peptide Z-Phe-Arg-pNA was added to a final concentration of 25 μM, and the enzyme/substrate mixture was incubated at room temperature.

In both cases (Fm-PEP and EP-B2), absorbance was measured at 410 nm every 5 min during a 5 h period. The mutant that showed enzyme-kinetics indicative of thermal stability was further analyzed. The kinetic parameters were calculated by measuring the initial velocity of the reaction, which was determined by the increase in absorbance at 410 nm. Initial velocities were plotted against substrate concentration, and *K*_*M*_ and *k*_cat_ values were calculated. The activity was calculated using the Beer-Lambert equation (A = ε c l), and the concentration of the product was calculated based on the extinction coefficient for pNa (8.8 mM).

A more detailed analysis was performed on the mutants that showed better performance in the initial screen. For such mutants, large-scale expression cultures in 60 ml LB medium were performed to obtain adequate quantities of the enzyme variants. After expression, the enzyme variants were retrieved in inclusion bodies, purified, and refolded. The kinetic parameters were calculated by measuring the initial velocity of the reaction, which was determined by the increase in absorbance at 410 nm. For making these calculations, the enzyme concentration was kept constant at 0.02 μM, and the substrate concentration ranged between 0.075 and 0.3 mM. Initial velocities were plotted against substrate concentration, and the slopes in each case were used to calculate *K*_*M*_ and K_*cat*_.

### Enzyme Performance Assay Using Wheat Gluten Proteins

After the characterization of enzymes using a synthetic substrate, activity was measured against wheat gluten standard procured from the National Institute of Standards & Technology (Canada). To study the effect of digestion on individual prolamin groups, 25 mg of gluten reference material was fractionated into gliadins and glutenins using the stepwise gluten extraction procedure described in Wen et al. (20). Digestion of gliadins and glutenins was performed stepwise, as described below. First, the gliadin and glutenin fractions at the final concentration of 25 mg/ml, were digested with pepsin (0.6 mg/ml) and pre-activated EP-B2 (0.375 mg/ml) under simulated gastric conditions (50 mM sodium acetate buffer, pH 4.5) for 60 min. The tube was incubated with gentle shaking (50 rpm) at 37°C. Second, pH of the solution was adjusted to 6.0 with 500 mM sodium phosphate buffer, and intestinal proteases, trypsin (0.375 mg/ml), chymotrypsin (0.375 mg/ml), elastase (0.075 mg/ml), and carboxypeptidase A (0.075 mg/ml) were added to the solution. Third, the prolyl endopeptidase variant (pre-exposed to 90°C for 10 min) was added to the solution at a concentration of 0.375 mg/ml, and the mixture was incubation at 37°C for 60 min with gentle shaking. Finally, the reaction was stopped by incubation of the tubes for 10 min at 100°C. Later, to clarify the solution, digests were centrifuged for 10 min at 9,300 g and filtered through 0.45 μm filters. Tricine-PAGE was used to analyze the digestion product using the protocol described in Schagger (51). After electrophoresis, each gel was subject to densitometric analysis using a Personal Densitometer SI, Model 375A (Molecular Dynamics), and the resulting images were analyzed using ImageJ software v 1.47.

### Reverse-Phase High-Performance Liquid Chromatography (RP-HPLC)

The HPLC separations were performed using a C8 reversed-phase analytical column (Zorbax 300SB-C8, Agilent Technologies) with 5 μm particle size and 30 nm microporous silica diameter (250 mm length, 4.6 mm inner diameter) and a C18 reversed-phase analytical column (Eclipse Plus C18, Agilent Technologies) with 5 μm particle size, 150 mm length, and 4.6 mm inner diameter. Both column types were used on a 1200 Series Quaternary HPLC-System (Agilent Technologies) with a diode array UV-V detector. During the runs, the column temperature was maintained at 60°C. A linear elution gradient was implemented using two mobile solvents, the polar solvent A consisting of 0.1% trifluoroacetic acid (TFA) (vol/vol) in type I ultrapure water (18 MΩ·cm specific resistance), and the non-polar solvent B containing 0.1% TFA (vol/vol) and acetonitrile (ACN). Absorbance was monitored at a detection wavelength of 210 nm, and the flow rate was maintained at 1.0 ml min^−1^ on the C8 column and 0.5 mL min^−1^ on the C18 column. In the case of C8 column, the elution gradient conditions were selected as follows: for gliadins, a linear gradient from 20 to 60% B in 60 min, and for glutenins, from 0 to 24% B in 20 min followed by 24–60% B in 40 min. After each run, the column was cleared with 90% B for 3 min and equilibrated with the starting B concentration for 5 min. In the case of the C18 column, the elution gradient condition was: a linear gradient from 0 to 50% B in 30 min. After each run, the column was cleared by linearly decreasing solvent B to 0% in 4 min, and then the column was equilibrated with the starting B concentration for 10 min.

## Results and Discussion

### Multiple Sequence Alignment and Phylogenetic Analysis of the Prolyl Endopeptidase Family

Prolyl endopeptidases can be found in archaeal, bacterial, and eukaryotic species (52). But for the purpose of this study, enzymes were selected for further analysis from the thermophilic organisms (Table S2). The BLASTP searches were performed against the NCBI nr database using the Pfu-PEP sequence as a query, and 16 PEP sequences from thermophilic organisms were identified. The range of survival temperatures for these thermophiles varied from 45 to 100°C. Among these thermophiles, members of the *Pyrococcus* family thrive at a temperature above 80°C and hence being classified as hyperthermophile or extremophiles. The alignment of the protein sequence was achieved using ClustalW, and as expected, a significant similarity between the protein sequences was observed (Figure S2). In the catalytic domain (between residues 1–73 and 428–710), 49 residues were 100% conserved among these sequences. Whereas, in the β-propeller domain, only two residues were 100% conserved, which is not surprising, because, in the *Pyrococcus* family, the β-propeller was not reported to be involved in the filtering of peptides large than the optimal substrate size (53). Thus, due to lack of selection pressure, it was expected to evolve faster than other parts of the enzyme, which explains the observed levels of diversity (52). Considering the high levels of diversity in the β-propeller domain, it is very likely that the variations contributing to the differences in the thermostabilities of different PEPs also lie in this domain. The observed level of similarity ranged from 22.28 to 41.11% between pairwise comparisons of different enzymes with FmPEP. The lowest level of homology ranging from 22.28 to 27.76% was observed between the prolyl endopeptidases from *F. meningosepticum* and *Pyrococcus*/*Thermococcus* families. Interestingly, when the optimum growth temperatures for organisms used to obtain these sequences were checked, a decrease in growth temperature below 80°C was found associated with the increase in percentage homology in sequences. This trend persisted up to 41.11% sequence similarity, as was observed in the comparison between Fm-PEP with *Deinococcus radiodurans* PEP.

The conservation of residues at the substrate-binding site was also studied (Table S3). The catalytic triad and the specificity pocket S1 showed complete conservation. The similarity between residues decreases in the case of the specificity pocket 3 (S3), because of its overlapping location with the β-propeller domain. At this site conservation of the residues varied between 27.8 and 77.8%. These changes have an influence on the activity of the enzyme and its substrate-binding properties (52). An example of such variability is residue 252. In the case of Fm-PEP, this amino acid location is occupied by tyrosine, whereas, in the PEPs of the *Pyrococcus* family, the same position is held by phenylalanine. In the case of *Pyrococcus* PEP, this change in the amino acid residue was found to be associated with the low turnover of the enzyme under experimental conditions, which might also be the case for other archaeal PEPs carrying phenylalanine at amino acid (aa) location 252. Not all PEP sequences derived from the thermophilic organisms have phenylalanine at position 252 and were also documented to have high cleavage efficiencies. It has been reported that members of the *Pyrococcus* family have the ability to hydrolyze proteins like azocasein (23.6 kDa), which was rather surprising for a prolyl endopeptidase. These observations led to the conclusion that the β-propeller domain of *Pyrococcus* PEP favors it opening on exposure to high temperatures, which also contributes to its autoproteolytic properties (54, 55).

The multiple sequence alignment of 16 PEPs formed the basis of constructing a phylogenetic tree using the distance- and character-based methods (Figure S3). The high conservation between the PEPs derived from the hyperthermophilic organisms suggested a close common ancestry. It is possible to draw conclusions about the structure-function relationships of PEPs by looking at the phylogenic relationships of the analyzed thermophiles. Also, it is possible to assume that the adaptation to different environmental conditions might have favored the selection of specific changes in the sequence of the β-propeller domain. The same is also true for the observed conservation of residues specific for the catalytic activity. An example of this situation is the high sequence similarity observed between the Fm-PEP and the PEP sequences of the *Deinococcus* family, and the fact that both bacterial species are adapted to mild environmental conditions.

### Site-Directed Mutagenesis and Analysis of Thermostability of Enzyme Variants

#### *Flavobacterium meningosepticum* Prolyl Endopeptidase (Fm-PEP)

Conserved amino acid residues from the β-propeller domain in the *Pyrococcus* family were selected for mutagenesis (Table 2). Nine sites were identified to introduce point mutations leading to amino acid substitutions in Fm-PEP, to create a library of 59 enzyme variants with differences in number and order of substitutions (Table S4).

**Table 2:** List of amino acid residues selected for introducing sequence guided substitutions in *Flavobacterium meningosepticum* prolyl endopeptidase gene sequence.

**Organism**	**Optimum growth temperature (°C)**	Amino acid locations^*^								
		**123**	**125**	**136**	**137**	**406**	**412**	**413**	**414**	**415**
*Sulfolobus tokodaii*	80	L	Y	K	T	S	R	V	V	K
*Metallosphaera sedula*	75	L	R	N	I	S	T	I	S	R
*Thermococcus barophilus*	48–95	I	L	K	L	S	R	L	Y	R
*Thermococcus sibiricus*	60–84	I	V	T	L	S	R	L	Y	K
*Thermococcus kodakarensis*	86	I	W	A	L	S	R	L	Y	Q
*Thermococcus onnurineus*	80–90	I	W	R	L	S	R	L	Y	E
*Thermococcus gammatolerans*	88	I	W	E	L	S	R	L	Y	E
*Pyrococcus yayanosii*	98	I	W	K	L	S	R	L	Y	E
*Pyrococcus furiosus*	100	I	W	E	L	S	R	L	Y	E
*Pyrococcus horikoshii*	98	I	W	E	I	S	R	L	Y	E
*Pyrococcus abyssi*	102	V	W	E	L	S	R	I	Y	E
*Aciduliprofundum boonei*	70	V	N	D	L	S	R	L	Y	E
*Sphaerobacter thermophiles*	65	L	E	P	N	S	T	V	F	Q
*Haladaptatus paucihalophilus*	45	V	D	P	N	S	T	V	Y	R
*Deinococcus radiodurans*	Variable	T	D	P	N	S	R	P	Y	R
*Deinococcus maricopensis*	45	V	D	A	N	S	T	P	H	H
*Flavobacterium meningosepticum*	37	R	D	P	N	N	T	I	F	K
*Natranaerobius thermophiles*	57	F	E	P	N	T	T	I	L	R

* *Conserved amino acid residues are highlighted in red font*.

Residues 123 and 125 were, respectively mutated from Arginine and Aspartic acid to Isoleucine and Tryptophan. In this case, the arginine to isoleucine substitution, favored interaction by van der Waals forces with the residues in proximity. On the other hand, the substitution of Aspartic acid to Tryptophan, a non-polar and hydrophobic amino acid, resulted in increased interactions by the formation of hydrogen bonds, aromatic stacking, and increase of van der Waals force.

The second set of substitutions was made at amino acid residues Proline and Asparagine located at positions 136 and 137. In this case, the first amino acid substitution of Proline to Glutamic acid, which can interact ionically, showed the formation of four hydrogen bonds and also van der Waals interactions. The amino acid residue at position 137 was substituted with Leucine. This change did not affect the flexibility of the peptide chain but enhance van der Waals interactions. Leucine does not form hydrogen bonds, because of the conformation of its side chains that are arranged in “L” shape in contrary to the “Y” shape of side chains in Isoleucine, which is often found at the core of protein folds.

The first mutation on the seventh blade was the change of residue 406 from Asparagine to Serine. In this case, the hydroxyl group of Serine conferred hydrogen bonding potential, and this amino acid substitution also increased the possibility of van der Waals interacts with close by residues.

The amino acid substitution at residue 412 from Tyrosine to Arginine increased the possibility of forming hydrogen bonds at seven potential sites and also increased the chances of ionic and van der Waals interactions. The next amino acid substitution introduced at residue 413 from Isoleucine to Leucine also increased the flexibility of interactions, especially by van der Waals forces.

The last couple of amino acid substitutions were introduced at positions 414 and 415. In the first case, Phenylalanine was replaced with Tyrosine, which potentially formed three hydrogen bonds and interacted by aromatic stacking as well as van der Waals forces. And as of the last mutation, Lysine residue at position 415 was replaced by Glutamic acid.

After the successful cloning of the mutants in the expression vector, DNA sequencing was performed to assure that the mutations were successfully introduced in the gene sequence. Initial screening of mutants was performed using a synthetic substrate to evaluate their activity after heat shock. Out of the 59 evaluated mutants, five showed significantly higher activity than the control at 80°C (Table S4). Mutations stacked at the amino acid positions 123, 125, 136, and 137 showed a detrimental effect on the stability of the enzyme at 80°C. Albeit, a combination of mutations involving residues at 412–415 showed stability at higher temperatures likely due to the formation of the hydrogen bond, witnessed in the *in-silico* structural analysis of mutants (Figure S4).

Based on the initial thermal analysis, five mutants, namely Fme5, Fme6, Fme10, Fme16, and Fme18, were exposed to 90°C for 10 min. After heat-shock, enzyme activity was measured, and only three mutants (Fme5, Fme6, and Fme10) retained their ability to cleave the synthetic substrate. Therefore, these mutants were selected for enzyme kinetics analysis. After heat shock at 60 or 90°C, *k*_cat_, *K*_*M*_, and *k*_cat_/*K*_*M*_ values were determined for each Fm-PEP variant (Table 3). A combination of mutations involving amino acid residues at 412, 413, 414, and 415 increased thermostability of the enzyme (Table 3).

**Table 3:** Kinetic parameters of Fm-PEP variants determined using a synthetic substrate, Z-Gly-Pro-pNA.

**Enzyme**	**60^°^C *k_cat_*/min^−1^**	**90^°^C *k_cat_*/min^−1^**	**60^°^C *K*_*M*_ (μM)**	**90^°^C *K*_*M*_ (μM)**	**60^°^C *k_cat_*/*K*_*M*_**	**90^°^C *k_cat_*/*K*_*M*_**
Wild type^a^	60.3898	2.9004	0.2034	0.1135	296.9157	25.5503
Fme5^b^	14.7133	7.49034	0.09896	0.1113	148.6823	67.3192
Fme6^c^	18.5709	5.4641	0.09655	0.0861	192.3406	63.4818
Fme10^d^	4.8203	5.8314	0.0418	0.1419	115.3152	41.0920

*Enzymatic activity was measured after a heat-shock at 60 and 90°C for 10 min. Absorbance at 410 nm was recorded. The Beer-Lambert equation (A = ε c l) and the concentration of the product was calculated based on the extinction coefficient for pNa (8.8 mM)*.

a *Corresponds to wild type prolyl endopeptidase from Flavobacterium meningosepticum used as a negative control*.

b *Corresponds to variant Fme5 having mutations at residues 412 and 413*.

c *Corresponds to variant Fme6 having mutations at residues 414 and 415*.

d *Corresponds to variant Fme10 having mutations at residues 406, 412, 413, 414, and 415*.

#### *Hordeum vulgare* Endoprotease B2 (EP-B2)

**Figure 2:**
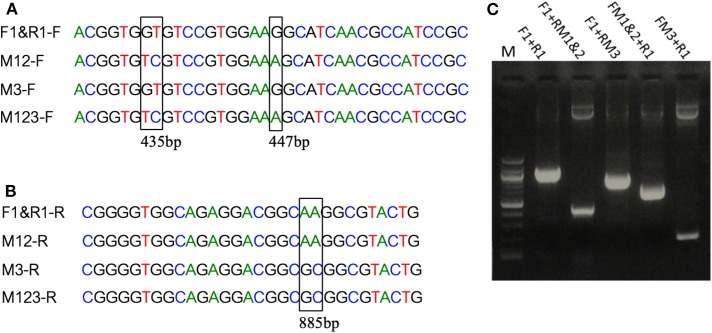
Introduction of mutations in EP-B2 by error-prone PCR. **(A,B)** Results of DNA sequencing showing the location of the desired point mutations (marked by a rectangle in the sequence alignment). M1 = GT to TC transversions, M2 = G to A transition and M3 = A to G transition, M12 represents a combination of M1 and M2, and M123 a combination of M1, M2, and M3. F1&R1 represents the wildtype sequence. F and R in the sequence names designate DNA sequences in Forward (+) and Reverse (–) orientations. **(C)** Agarose gel analysis of the PCR products obtained using various combination of primers (for primer sequences, see Table S1). Different sets of primers were used in PCR to obtain different combinations of mutations and thus various EP-B2 variants.

**Figure 3:**
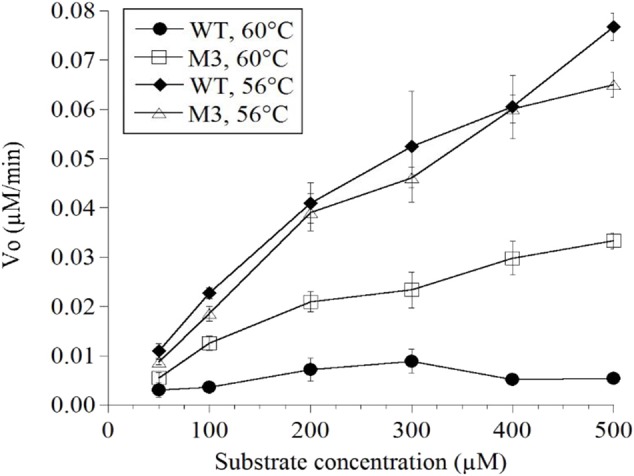
Determination of the thermostability of wild type (WT) control and EP-B2 variant dubbed M3 (K180A) using a synthetic substrate, Z-Phe-Arg-pNA. Enzyme activity was measured at 56 and 60°C, and was compared with the wild type control. To monitor enzyme activity absorbance was recorded at 410 nm. The Beer-Lambert equation (A = ε c l) and the concentration of the product was calculated based on the extinction coefficient for pNa (8.8 mM). Concentration is expressed as μM/min.

Mutations in the EP-B2 gene sequence were introduced as described earlier, and the point mutations were confirmed by DNA sequencing (Figure 2). SDS-PAGE was used to confirm the expression of EP-B2 in *E. coli* (strain BL21) and *in-vitro* enzyme activity assay using a synthetic substrate, Z-Phe-Arg-pNA to confirm refolding as well as activation. The effect of temperature on enzyme activity was also determined using synthetic substrate, Z-Phe-Arg-pNA via spectrophotometry (Figure S5). The heat-shock analysis of the EP-B2 variants (with one, two, and three mutations) suggested that the enzyme variant with a single substitution at amino acid location 180, dubbed M3 (K180A), retained activity up to 60°C (*T*_max_). In comparing with the wild type, the mutant showed an increase in thermostability by 4°C. On the other hand, the *T*_max_ of M1&2 and M1&2&3 did not show a noticeable improvement. In fact, the activities of the M1&2 (V34S and G38S) and M1&2&3 (V34S, G38S, and K180A) were slightly lower than the wild type. This could be explained because of amino acid substitutions affected protein structure, disturbing binding sites, and thus reducing catalytic activity. Although the *T*_max_ of M3 was higher than the control, the activity of the enzyme declined with the increase in temperature, as can be witnessed in Figure 3 and Table 4. In sum, site-directed mutagenesis has the potential to increase EP-B2 thermostability. However, the 4°C increase in thermostability observed for the M3 (K180A) variant is not sufficient, and a further increase in it is needed, which could be achieved probably by the next round of directed evolution.

**Table 4:** Kinetic parameters of EP-B2 variant “M3” and the wild type (WT) control was determined using a synthetic substrate, Z-Phe-Arg-pNA.

	**M3, 56°C**	**M3, 60°C**	**WT, 56°C**	**WT, 60°C**
*V*_max_ (μM/min)	0.236	0.0506	0.142	0.00746
*K*_*M*_ (μM)	1188	318.3	530.0	104.0

*Enzymatic activity was measured after a heat-shock at 56 and 60°C for 10 min. Absorbance was recorded at 410 nm. The Beer-Lambert equation (A = ε c l) and the concentration of the product were calculated based on the extinction coefficient for pNa (8.8 mM)*.

### Testing the Activity of Thermostable Fm-PEP Variants Using Gliadins and Glutenins

**Figure 4:**
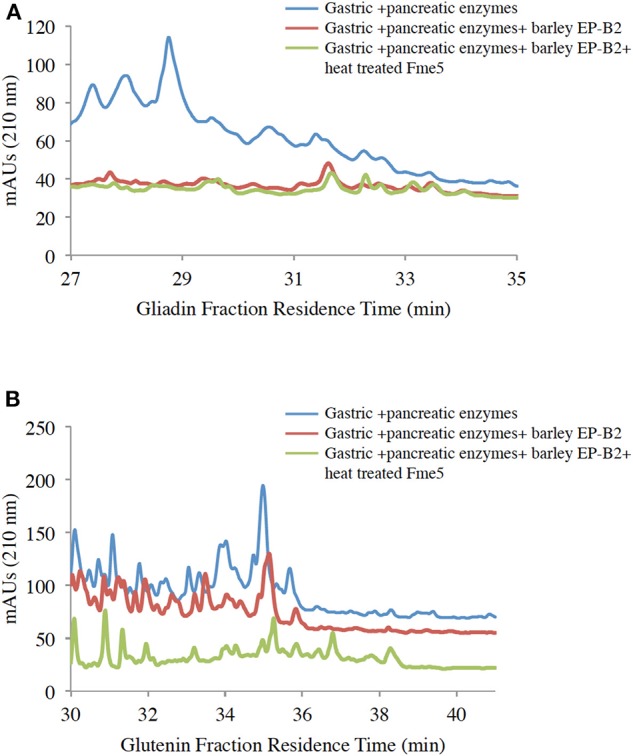
RP-HPLC (reversed-phase high-performance liquid chromatography) analysis of wheat gliadin **(A)** and glutenin **(B)** fractions. Before HPLC, gliadins were sequentially treated with digestive enzymes (pepsin, trypsin, chymotrypsin, elastase, and carboxypeptidase **(A)**, EP-B2, and Fm-PEP variant “Fme5.” Subsequently, pancreatic enzymes were added to the solution after pH adjustment. The control was wheat gliadins digested only with gastric and pancreatic enzymes (blue line), and the treatments were wheat gliadin digestion with gastric and pancreatic enzymes supplemented with (i) EP-B2 (red line), or (ii) EP-B2 and heat-treated (90°C for 10 min) Fm-PEP (green line).

To further affirm the activity of the thermostable Fm-PEP variants against the intended substrate after heat shock treatment, the enzyme was supplied with gluten protein fractions under simulated gastrointestinal conditions. For this purpose, the prolamins were extracted as described in materials and methods, and the resultant protein fractions were used as the substrate for the enzymatic digestion. Each fraction, gliadin and glutenin was subject to three treatments: (i) Gastric and pancreatic enzymes (pepsin, trypsin, chymotrypsin, elastase, and carboxypeptidase A); (ii) Gastric and pancreatic enzymes plus activated EP-B2; and (iii) Combination of gastric and pancreatic enzymes, activated EP-B2, and either of the three heat-treated Fm-PEP variants or its wild type version (negative control). And after each treatment, the digests were analyzed by RP-HPLC on the C18 column. Comparative analysis of the treatments comprising only gastric enzymes with the one with the addition of glutenases (Figure 4A) showed evidence of gliadin degradation, where degraded peptides appeared between 18 and 24 min elution time on chromatograms, with only a small amount of peptides (potentially immunogenic hydrophobic large peptides) appeared after 30 min elution time. The combination of EP-B2 (wild type-control) and heat-treated Fm-PEP variant “Fme5” successfully detoxified the gluten reference material. It suggested that essentially, the enzymatic treatment with glutenases has detoxified the majority of the immunogenic peptides, based on the fact that large hydrophobic peptides elute late when the column conditions become more non-polar. The results of the analysis showed correspondence with the earlier studies (29), which showed gluten degradation to ~28-mer peptides by the addition of EP-B2. It was also shown that the most immunogenic gluten peptides elute around 22 min (56) using a similar gradient of polar and non-polar solvents in RP-HPLC on the column type similar to the one used in the present study. These earlier studies further support our conclusion that the thermostable variants of Fm-PEP retain the capability to degrade immunogenic peptides after heat-treatment.

The glutenin fraction was also analyzed (Figure 4B) using RP-HPLC. Reduction in the content of immunogenic peptides appearing between 28 and 35 min in chromatograms of EP-B2 or EP-B2 plus Fm-PEP (variant) treated glutenin samples was observed. These observations were supported by the results of Gass et al. (30), who reported complete degradation of immunogenic peptides by sequential treatment of gluten samples with EP-B2 and *Myxococcus xanthus* PEP under simulated gastric conditions.

**Figure 5:**
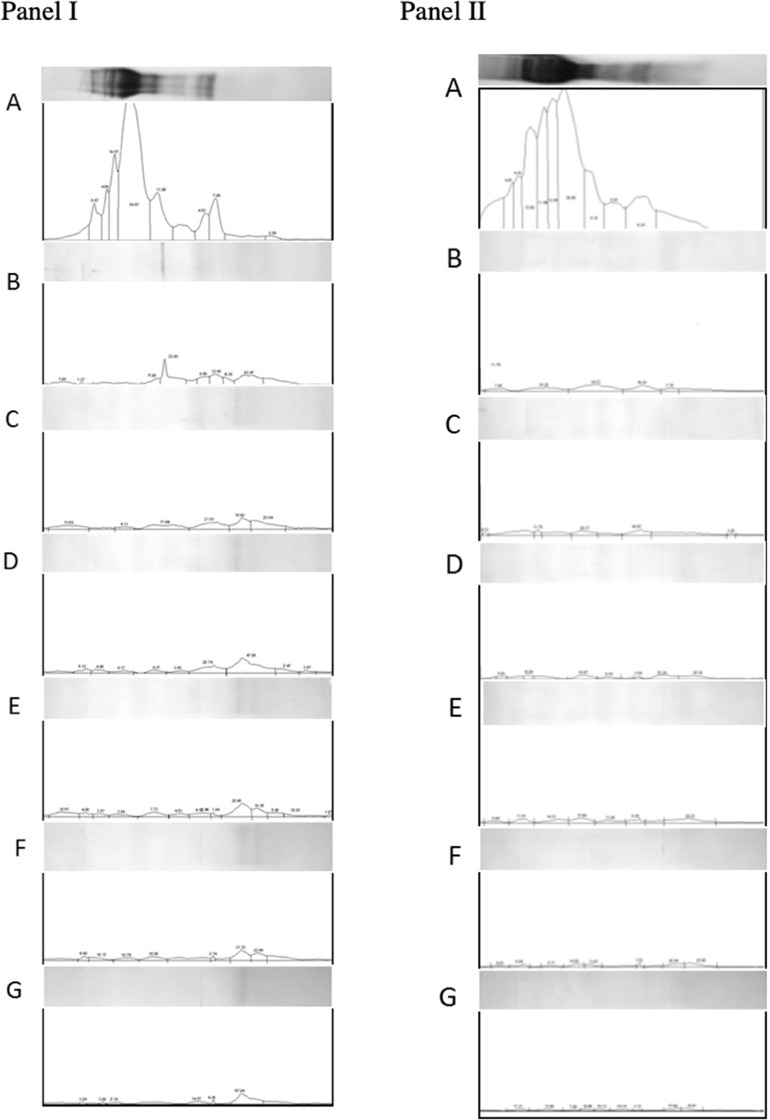
Densitometric analysis of gliadin (panel I) and glutenin (panel II) fractions loaded on Tricine-PAGE. **(A)** Undigested gliadins/glutenins (negative control). **(B)** Gliadins/glutenins digested with gastric and pancreatic enzymes. **(C)** Gliadin/glutenin fraction digested with gastric-pancreatic enzymes in combination with barley cysteine endoprotease B2 (EP-B2). **(D)** Gliadin/glutenin fraction digested with gastric-pancreatic enzymes, EP-B2, and native *Flavobacterium meningosepticum* prolyl endopeptidase (Fm-PEP) (positive control). **(E)** Gliadin/glutenin fraction digested with gastric-pancreatic enzymes, EP-B2, and Fm-PEP variant, Fme5. **(F)** Gliadin/glutenin fraction digested with gastric-pancreatic enzymes, EP-B2, and Fm-PEP variant, Fme6. **(G)** Gliadin/glutenin fraction digested with gastric-pancreatic enzymes, EP-B2, and Fm-PEP variant, Fme10.

Densimetric analysis of digested gluten proteins resolved on tricine-PAGE gels also supported the observations made using RP-HPLC. Based on cumulative area estimations under each peak on the densitogram and using a BSA standard curve as a reference (57), amounts of loaded proteins were determined. Digestion with gastric and pancreatic enzymes resulted in a decrease in the amount of proteins from 344.54 to 60.69 μg/ml. Similarly, the addition of EP-B2 decreased the amount further to 48.08 μg/ml. The addition of native Fm-PEP did not result in any further decrease in the concentration (up to 48.08 μg/ml). Interestingly, the addition of prolyl endopeptidase variants numbered 5, 6, and 10 (Fme5, Fme6, and Fme10) resulted in a reduction of protein amount from 48.08 μg/ml to 25.54, 17.61, and 20.28 μg/ml, respectively (Figure 5, panel I). However, in the case of the glutenin fraction, no difference in terms of protein amount could be detected by the addition of prolyl endopeptidase in the fraction (Figure 5, panel II). These observations correspond well with the results of *in silico* analysis. Specifically in terms of the activity of endopeptidase on different gluten fractions, showing better cleavage of the gliadins than glutenins. It could be explained in terms of the size of the peptides produced after EP-B2 treatment, which makes them suitable for PEP-activity. In sum, these results suggested that Fm-PEP variants retain their activity after a heat-treatment at 90°C for 10 min and are capable of detoxifying the gluten protein to near-completion. This property will add value to the glutenases, as it will allow their expression in grain and use of such grains in the food industry without loss of enzyme activity due to thermal denaturation.

## Conclusions

Since a combination of prolyl endopeptidase and barley cysteine endoprotease has been proven to be effective in detoxifying gluten proteins, efforts were made to increase their thermostability, to make them suitable for industrial applications. Site saturation mutagenesis effectively increased the thermostability of Fm-PEP, which should be sufficient to maintain the activity of the enzyme in the core of bread, where temperatures generally do not exceed 100°C. Given, accumulation of enzymes in protein storage bodies in endosperm cells and the use of “glutenase” expressing grains as such or cracked grains in bread is expected to provide further insulation from thermal denaturation. On the other hand, directed mutagenesis of EP-B2 resulted in the limited increase in the thermostability of the enzyme, however insufficient in combination with the inherent properties of the EP-B2 pro-peptide as a molecular chaperone and its accumulation in protein storage bodies is expected to provide further encapsulation and resistance from melting. Still, a further increase in the thermostability of EP-B2 is desirable to seek full advantage of the complimentary digestive properties of Fm-PEP and EP-B2 on gluten detoxification, especially in processed foods. Therefore, research is being conducted to mutagenize the gene encoding EP-B2 further. Once both thermostable enzymes have been developed, wheat grains expressing a combination of these glutenases could be produced, which will constitute an alternative for the treatment of celiac disease.

## Data Availability Statement

All datasets generated for this study are included in the article/Supplementary Material.

## Author Contributions

CO, SR, and DW contributed the conception and design of the study. CO, NW, JM, SM, and SR performed the experiments. CO and SR wrote the first draft of the manuscript. SR edited the manuscript.

### Conflict of Interest

The authors declare that the research was conducted in the absence of any commercial or financial relationships that could be construed as a potential conflict of interest.
