# Supplementary material for: Directed-Mutagenesis of Flavobacterium meningosepticum Prolyl-Oligopeptidase and a Glutamine-Specific Endopeptidase From Barley
Source: Front Nutr. 2020 Feb 18;7:11. doi: 10.3389/fnut.2020.00011 (PMC7040222; doi:10.3389/fnut.2020.00011)
Supplement: Supplementary file 1 [file Data_Sheet_1.docx]

Supplementary Material

**Text S1**. **Determination of the optimal temperature for the activity of *Hordeum vulgare* EP-B2**

Successful expression of EP-B2 was followed by activation of the enzyme at the acidic pH using acetate (pH 4.5) and sodium citrate (pH 3.0) buffers. Activation of the enzyme was confirmed by loading it on the 12% SDS polyacrylamide gel, and its activity was measured using a synthetic substrate (Figures S1A and B). The highest activity was achieved when the acetate buffer was used for activation of the pro-enzyme to active EP-B2. Kinetic parameters were calculated to quantify the efficiency of the active enzyme under normal conditions, obtaining a *V*_max_ of 0.071088 µM/min, *K_M_* of 138.0323 µM, and *k*_cat_ 0.236961 min^-1^. In order to test the stability after heat-shock, pro-enzyme was exposed to 60ºC for 10 minutes. And no detectable activity was observed after the heat treatment. The experiment was replicated thrice (Figure S1C). It can be concluded from the above experiment that temperatures ≥60°C completely defunctionalize EP-B2. Although based on the 3D-structure of the pro-enzyme, protective function for the pro-peptide was suggested, and the refolding of the enzyme after its thermal denaturation was proposed (Cambra et al., 2012).

**References**

Cambra, I., Hernandez, D., Diaz, I., and Martinez, M. (2012). Structural Basis for Specificity of Propeptide-Enzyme Interaction in Barley C1A Cysteine Peptidases. *Plos One* 7(5). doi: 10.1371/journal.pone.0037234.

**Figure S1.** *Hordeum vulgare* cysteine endoprotease (EP-B2): activation and kinetic analysis under different temperature conditions. A) Activity of barley cysteine endoprotease using a synthetic substrate, Z-Phe-Arg-pNA after activation with acetate buffer (blue diamond) or sodium citrate buffer (crimson square). B) SDS-PAGE analysis of proEP-B2 and EP-B2 after activation. Notice cleavage of the propeptide using two different buffers. C) Activity of EP-B2 measured using a synthetic substrate Z-Phe-Arg-pNA under room temperature (blue diamond) and after the heat-shock at 60ºC for 10 minutes (crimson symbol).


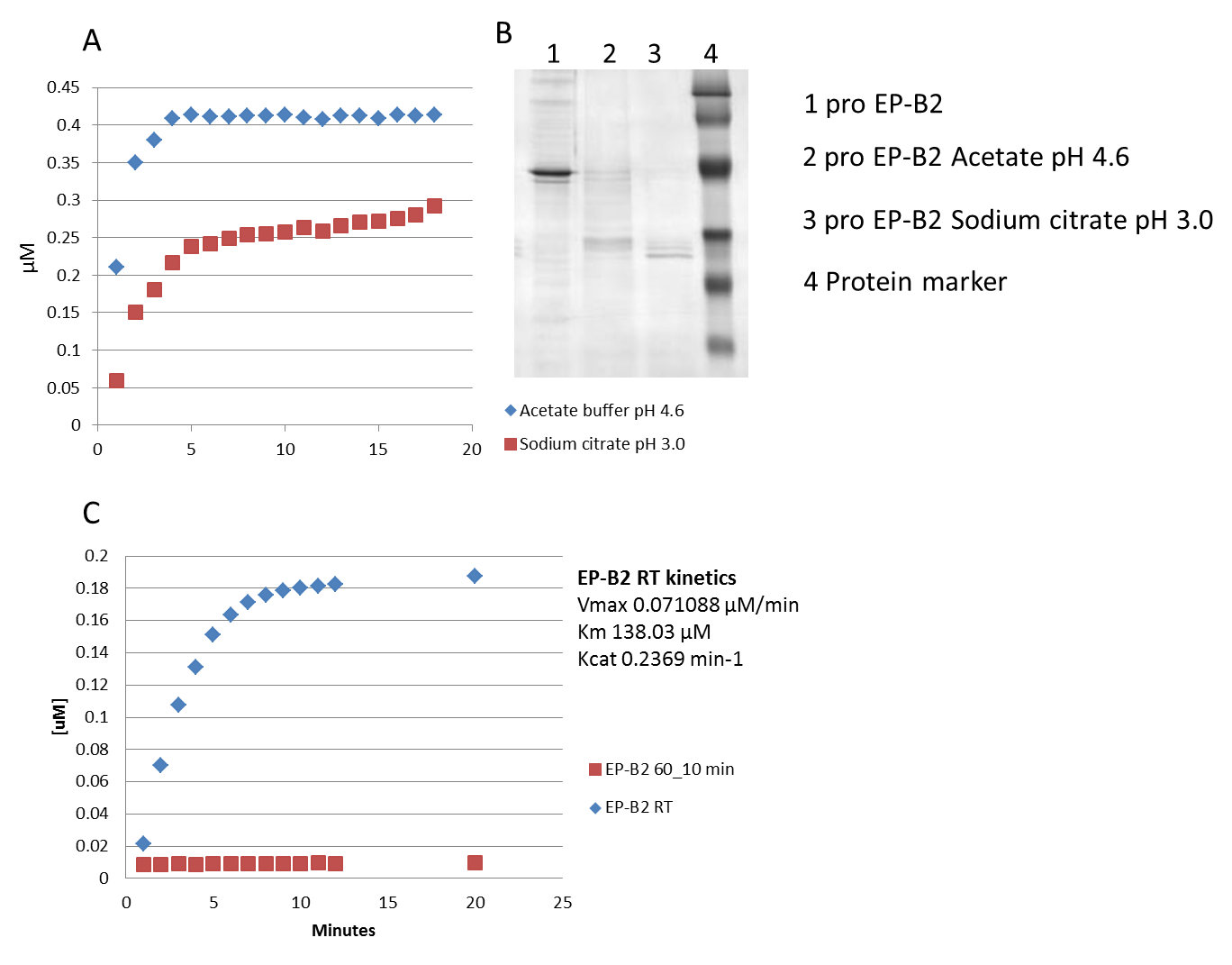


**Figure S2.** Multiple sequence alignment of prolyl endopeptidase sequences, derived from thermophilic organisms, performed using ClustalW (<http://www.ebi.ac.uk/Tools/msa/clustalw2/>). Sequences were retrieved from NCBI.

Sul.tokoda ---------------------------------------------------MDEYEYLEN

M.sedula ---------------------------------------------------MDPFEYIEN

T.barophil --------------------------------------------------MEDPYLWMEN

T.sibiricu -----------------------------MLKQMSDTLINVKSIILMVILMEDPYIWMEN

T.kodakare --------------------------------------------------MEDPYIWMEN

T.onnurine --------------------------------------------------MDDPYMWMED

T.gammatol --------------------------------------------------MEDPYIWAEN

P.yayanosi --------------------------------------------------MEDPYLWMEN

P.furiosus --------------------------------------------------MEDPYIWMEN

P.horikosh -------------------------------------------------MVEDPYIWMEN

P.abyssi --------------------------------------------------MEDPYIWMEN

A.boonei ---------------------------------------------------MDPYEWMEN

Sp.thermop ---------------------MTERLTSPPATRVEPVTEVLHG-----HTIVDPYRWLED

H.paucihal ------------------------MLRTPPPTETRDVVETLHG-----EEIHDPYRWLEG

D.radiodur -----------------------MSKPYPQSPRGD-HVDVYQNAAGQEVRVPDPYRWLED

D.maricope ------------------MNNQELTGAAPAARRTE-HVDEYHG-----ERVPDPYRWLED

Sus.scrofa ----------------------MLSFQYPDVYRDETAIQDYHG-----HKVCDPYAWLED

F.meningos MKYKKLSVAVAAFAFAAVSAQNSNSLKYPETKKVN-HTDTYFG-----NQVSDPYRWLED

N.thermoph ----------------------------MSSKNSRIVVEDFHG-----TKVYDPYRWLED

* : : *.

Sul.tokoda LSDPRTISFIEKENKETESKLGKKAMELYP-------LLLEMDKEP--------YVLSMF

M.sedula LEDPRTKAFIEEETR-NSSFFQERAKLHYQ-------PILERLTEE--------RPITLV

T.barophil LNDERVLKFIEEENKRFREFIEDLPEKLID-------DVRKYYYLPNIWEAQITKRGTFV

T.sibiricu LTDERVLKFVNEENTRFREFIGELPDRLID-------KVKKYYYLPNIVEARITKKGTFV

T.kodakare LQDERVLKLVEEENRRFREFIGKLSDELFP-------EVWGLYSLPTLHSARLTEKGIIA

T.onnurine LQDERVLKLVEEENKRFREFIGELSDELFP-------EVWEYYSMPTLYGAKLTEKGIIV

T.gammatol LKDERVLKLVEEENKRFREFVGELSDELFP-------EVWEYYSIPTLHQVKLTERGIIG

P.yayanosi LSDERVLKLVEDENGRFRALVARLSDELYP-------EVKELYFLPIVRMARLTKKGTLV

P.furiosus LEDERVLKIIEEENKRFREFIGELSDKLFP-------EVWEQFSQPTIGMARITKKGIIA

P.horikosh LQDNRVREIIERENKKFREFIGELSDKLFP-------EVWEYFSIPTVTMAKITKRGIIV

P.abyssi LQDERVLKIVEEENRRFRELVGELSDKLFP-------EVWEYFSQPSIGMARITKRGIIV

A.boonei LEDERILKLVNEENKRFRDYVDGLSDKLAD-------EIRHYYYLPTIWEAKITKKGIIA

Sp.thermop DESPETRAWVDAQNAYTRRVLDASPSHARIRARLETLLSIGDISAPVMRGERAFFLRRE-

H.paucihal DDE-DVSAWVSAQNEYAKSFLRG-ETRDDLEPRFRALANS-AAYFPVVPTPNGYFQRYTP

D.radiodur PDSPETRRWVEAQNAVTEDFLAALPARAAYRERLTALWDYPRDGLPWERG-GRYFRTFNP

D.maricope AENAEISAWVDAQNAHTEAFLSASPDREAFRERLADLWDFPKPGTPWRRG-ARYFRTHND

Sus.scrofa PDSEQTKAFVEAQNKITVPFLEQCPIRGLYKERMTELYDYPKYSCHFKKG-KRYFYFYNT

F.meningos DRAEDTKAWVQQEVKFTQDYLAQIPFRGQIKKQLLDIWNYEKISAPFKKG-KYTYFYKND

N.thermoph EKAPEVKEWREREQEQTKNFLEG-DLKTKVKQRLEKLYSFPQLYIPVKKG-QRYFYQYHD

. : .

Sul.tokoda AYDEENPAILLYGEK-SQLLLGNKTIYIPPEGYVASEIWKVYNSKEIGVSIERKGSDKII

M.sedula G-TEKGVAILVRSKSGVHAEVNGNIIRSEREQDIFNSLERVWNSDLVRIGVGIGGSDQGY

T.barophil KINEAGRQLIKLLETGEIIVDSKKLEEELGDEILLQGFTVDRDGKRLAYNFSIGGADEGI

T.sibiricu KVNEGGKQIIKVLETNEVIITSETLEKELKDEILLQSFTVDEDGKRLAYNFSIGGSDEGI

T.kodakare VFKERDRQVIRWLN-GDVIVDSKALEAEIGDEVLLQGFTADGKGKRLAYSFSIGGADEGV

T.onnurine MFKERDRQIIKWLG-GKILVDSKRLEEELGDEVLLQGFTADDAGKRLAYSFSIGGADEGI

T.gammatol MYREGEGQVIRWLG-GDAIVDSKELEKELNDEVLLQGFTADRKGRFLAYSFSIGGADEGI

P.yayanosi MVNGAGKQMIRWFD-GEILVDSKKLEEEFGDEVLLQGFTADREGRKLAYSFSIGGADEGI

P.furiosus SYSEKDRVVIKWFN-GDVIVDSKELEREVGDEVLLQGFTTDEEGEKLAYSFSIGGADEGI

P.horikosh SYNEKDRSIIRWLD-GDVIVDSKEIEREVGDEVLLQGFTADDEGEKLAYSFSIGGADEGI

P.abyssi SYSEKDRVTVRWLG-GEVIVDSKELEKELNDEVLLQGFTTDEDGKRLAYSFSIGGSDEGI

A.boonei AINEHGKQVVRNLSSGEILIDSKDLEKELKDEILLQGFSVDEHLKFFAYNFSIGGSDEGI

Sp.thermop GYENQPKLYLREG-DSER-VLLDPNQESAEGTTALDWWYPSPDGTLVAFGYSQDGDEESV

H.paucihal ADEDLPLLTVRDDLDGERRVLADPNEWSDDATLTLDWFVPSPDGSLVAYGVTEGGTEQYD

D.radiodur GLLNQPVLQTADSPRGPWHELLDPNALSADGTVALMGASVSQDGTQLAYATQSGGSDWLT

D.maricope GLRNQPELQVADAPRGPWRTVLDANTLSDDGTVALMQVHVHHDGARLAYATQQGGSDWLT

Sus.scrofa GLQNQRVLYVQDSLEGEARVFLDPNILSDDGTVALRGYAFSEDGEYFAYGLSASGSDWVT

F.meningos GLQAQSVLYRKDAS-GKTEVFLDPNKFSDKGTTSLANLSFNKKGTLVAYSISEGGSDWNK

N.thermoph GLQNQPVLYFREANEDKEKLLVDPNKFSDDGTTAITVFFPSDDGKLLAYSLSRKGSDWQE

. * :

Sul.tokoda TLLIS-PEGK-IRELGEMVES----PFYFKGELCYIKSYRYSPPPDGGDYPADR------

M.sedula SILVN-EQGKVVRRVEGLVNQ----FFFLRGKLCYVREYRTESSPDGVPPAVER------

T.barophil TRIVNLETGEIIEEIKPSLWN----IVFLDDGYYFARFYRKEKTPDGISAPAER------

T.sibiricu TRIIDFENGKIVEEIKPSLWN----ITFLEDGYYFARFYRKEKTPDGIDAPAER------

T.kodakare TRIIDLESGELIDELRPSVWN----VTFLENGYYFSRFYRHGETPDGVKAPAVR------

T.onnurine TRIIDLETGELIDEMKPSVWN----VAFLENGYYFGRFYRHDETPDGVKAPAVR------

T.gammatol TRIVDLETGELLEEFKPSVWN----VTFLENGYYFARFYRHGETPDGVKAPAER------

P.yayanosi TRITDLESGELIEELRPSVWG----ITFLENGYYYVRFYRREKTPDGVKAPAVR------

P.furiosus TRIIDLKTGEVIEEIKPSIWN----ITFLKDGYYFTRFYRKEKTPDGVNPPAAR------

P.horikosh TRIIDLKSGEVIEEIKPSIWN----ILFLKDGYYFARFYRKEKTPDGVDPPAER------

P.abyssi TRIIDLETGELLEEIKPSVWN----IVFLDKGYYFARFYRKEKTPDGVNPPAER------

A.boonei TRVVNLESG-RVEEIKPSINN----IIFLRDGYYFSRFYRKQESPDGMKPPVER------

Sp.thermop LQVLDVARDTLLAERIDRTRFCSLAWLPDASGFYYTRYPQPGEVPPGEERYHRKVFFHRL

H.paucihal VRVVDAKTGDTVDELRETGRTGERSFAWSDEGFYYGR---TGALEDGGQ-LDKSLAYHRL

D.radiodur WQVRDVASGEDTGEPLRWSKFSGAAWLPDGSGFFYSAYDAPG--EGEALTGANKNQRLMF

D.maricope WRVRDVDTGEDLPDVVQWSKFSGAAWLPDGSGFLYSAYDAPA--SGEAYVSVNLHQKLYL

Sus.scrofa IKFMKVDGAKELPDVLERVKFSCMAWTHDGKGMFYNAYPQQDGKSDGTETSTNLHQKLYY

F.meningos IIILDAETKKQIDETLLDVKFSGISWLGD-EGFFYSSYDKPK--DGSVLSGMTDKHKVYF

N.thermoph IYIINVETGEKYPETIQYCRFTNVAWSKDNLGFYYSRFPNPE---GVAEEDQNKYNKVYY

. . :

Sul.tokoda -VFCK----DEIVYGKDMKPGEFVTIKVFDD--FLTLVRQKGWRYGELYVG--EGFD---

M.sedula -LFCG----EEMLPFYPGR-GEWISVKAEGD--NLLLVRGIGWSKKVLYR----------

T.barophil -IFLKKGEDEVMVSGEGFGSGYFMNLHKSTDGKWAMLTVTFGWNKADIYLGPIDEPE---

T.sibiricu -IFLKKGKEEKMVFGEGLGSGYFMSIQKTKDKKHALVTLSFGWNKNDIYFGPLSEPE---

T.kodakare -LFWKDESGERMVFGQGLGSGYFMGLRKSTDGKWAMLTVTFGWNRADIYIGPIEAPD---

T.onnurine -LFWKDEGGEKMVFGEGLSSGYFIGLRESTDGKWAMVVVTFGWNKAEIYLGPIEEPE---

T.gammatol -LFWKDESGEKIVFGEGLGSGYFISLGKSTDSKTAMVTVTFGWNSAEIYVGPIDEPE---

P.yayanosi -LFFRDEKGERMVFGKGLGSGYFISLRKSTDGKCAMLTVTFGWNSAEVYIGPIEEPE---

P.furiosus -MFWKDREGERMVFGEGLTSGYFMSIRKSSDGKFAIVTLTYGWNQGEVYIGPIDNPQ---

P.horikosh -VFWKDKEGEKMVFGEGLTSGYFMTLRKSSDDKFAMLTLNYGWNQGEIYVGPIDKPQ---

P.abyssi -IFWKDEEGERMVFGEGLTSGYFMSLRKSTDGKFAMLTLTYGWNKAEIYLGPIDKPE---

A.boonei -LFWRDKNGEKMVFGEGLDSNYFMYIKKSNDGKYAILTKTFGWNSSEIYLGPIENPE---

Sp.thermop ---GDDPAADPLVFGDGLPAEAQPHVRLSRDGRWLIVTVSHGWARADLYLHDRT------

H.paucihal DGGGEDDADDELPI--ELDEQTWAGLHTDPDSDYLVVELTRGWERTDVYAAR--------

D.radiodur HRLGTPQDADELVLERPDQPDWGFAAEVTEDGAWLAVQVWLGTSPKNLLWVRPLGKEGPG

D.maricope HRLGTPQADDELLLARPDQPEWGFEARIMRDTGRLIVSVWKGTLRQNLLWWRDLGADG--

Sus.scrofa HVLGTDQSEDILCAEFPDEPKWMGGAELSDDGRYVLLSIREGCDPVNRLWYCDLQQESNG

F.meningos HKLGTKQSQDELIIGGDKFPRRYLSGYVTEDQRYLVVSAANATNG-NELYIKDLKNKT--

N.thermoph HKIGRDQSNDELIYEDNHDKELVFNPFLTHDGEYICLFVRKGTDPRNGFYIKKADSED--

: : * : .

Sul.tokoda ------SLRKVDEGEV-IDVIDFQQGEVIYQKNNAVYLG--------------------N

M.sedula ------DFEKVDEGD--ITSYDMKGGRIYYVKGNSLMRD--------------------G

T.barophil ------KWRKVYSSDVPAHPIDVINGKLYIYTRKGRGLGKVIAIENG----------KVE

T.sibiricu ------KWKKVYSSEVPAHPIEYVNEKLYIYTREGRGLGKVIALENG----------ETR

T.kodakare ------RWEKVYSADVPAEPIDVVNGRLYILTREGKGLGKVIAIEGE----------KTI

T.onnurine ------KWEKVYSAEVPAQPIEVVDGKVYILTKEGKGLGKVIAIEDG----------EIE

T.gammatol ------KWEKVYSAEVPVEPVDVRDGTLYLLTKEGKGRGKLIAVMDG----------ELM

P.yayanosi ------KWEKVYSAGVPAEPIDVVNGKLYILTREGRGLGKVVAIKGG----------EVE

P.furiosus ------EWKKVYSASVPVEAIDVVNGKLYILTKEGKGLGKIIAIKNG----------KID

P.horikosh ------EWRKVYSATVPVEPVDIVNGKLYILTREGRGMGKVIAVKDG----------EIE

P.abyssi ------EWKKVYSADVPAEPIDVIDGKLYILTKEGKGLGKVIAVKDS----------EVE

A.boonei ------KWKKVYKSEVPAEPIDIVDGKLYVLTREGSGYGKIIAIG-E----------DIE

Sp.thermop -RPEAGFIPVFVEEEALVEGFVH-RGQLYLLTNLDAPRYRLLAVDPE----RPERQHWRE

H.paucihal ---DGEFEPLVAGEDAIFEPHLR-GNRAFFLTSLDAPNYRIVAAELADAAGRDAPETFES

D.radiodur SG---DFQPLVNDFQAMYQLVGSDGNTLFLQTDEDAPLGKLMAWNIR---TGE----RRD

D.maricope -----AFHELVGDFHASYDVVGMDGSTLYVLTDEDAPTGRLIAWDLS---TGE----RRD

Sus.scrofa ITGILKWVKLIDNFEGEYDYVTNEGTVFTFKTNRHSPNYRLINIDFT---DPEESKWKVL

F.meningos -----DFIPIITGFESNVGLVDTDGDTLFLHTDKNAPNMRMVKTTIQ---NPK-PETWKD

N.thermoph -----NFTKLFPQGEAMYKPMGIIGNTFYFLSDKQAPKGKIIAVDLN---NQT----QKT

. .

Sul.tokoda TKVVEVDYPVLGVSHIGDKIAVEVIKEYRTPLIFYDIKGKKIGEEVHDN---ITFMDGKG

M.sedula VELFKISRPTLDMKVMDDGILTLEIRNYKTSLVKYSEEGRETWNYTTDH---ILTFDTVG

T.barophil EIIPEDEFPLEWAVIVNDKILAGYLVHASSKLKVFTLEGELIDGISFKMPAQVYPLDNDG

T.sibiricu EIIPEGEFPLEWVIIVKDKILAAYLVHASSLLKVFTLDGELIEEIKFDMPGQVYPLDNNG

T.kodakare EVVPEGEFPLEWAVIVGDRILAGRLVHASHRLEVYSLNGEKLDEIAFDLPGSVYPLDSDG

T.onnurine EVIPEDEFPLEWAVIVGNRILAGRLVHASHRLEVYSLDGKKLDEITFDLPGSLYPLDADG

T.gammatol EIIPEGEFPLEWAVLVGDKILAGRLVHASHILEVYSLSGEKLGEITFDLPGSVYPLDTDG

P.yayanosi EIIPEGEFPLEWAVIVDDKILAGRLVHASHRLEVYSLGGEKLDEVTFDLPGSVYPLDIHA

P.furiosus EVIPEGEFPLEWAVIVRDKILAGRLVHASYKLEVYTLNGEKIKEITFDVPGSLYPLDKDE

P.horikosh EVIPEGEFPLEWAVIVDDKIVAGRLVHASHRLEVYTLKGEKIEEVTFDIPGALHPLDKNN

P.abyssi EIIPEGEFPLEWAVIVKDKILAGRLVHASHKLEVYNLKGEKISEVEFDFPGSLYPLDKDD

A.boonei EVVPEGEYPLEWAEIVNGKILAGYLVDASSKLKLFSLDGKEVKEIKFEPAGKVIPFDSKD

Sp.thermop IIAEPEEARIEQVVPVGDRLVVQTLVRATSRLTLHDLDGRPVSTLDLPGLGTVS--GLNA

H.paucihal VVPERDDAILRDFVLAGDHIVAHYERDAVSELVAFDLDGTRAEDASLPELGSIDEGSLHG

D.radiodur LLPEGADKLEQVLTVPGGFLALT-LHDASHRLTLYDRNGERQREIELPALG-TVSVSAEQ

D.maricope VIAASADTLHTVAVARDALVTVS-LRDARDRLVIHDRRGTPLREVHLPDLASVLEVNATA

Sus.scrofa VPEHEKDVLEWVACVRSNFLVLCYLHDVKNTLQLHDLATGALLKIFPLEVGSVVGYSGQK

F.meningos VIAETSEPMR--VNSGGGYFFATYMKDALSQIKQYDKTGKLVREIKLPGSGTAGGFGGEK

N.thermoph VIAETDKIISDAAVIN-NHLVLVYQDHGSHLVNIFNLDGVKVDQITLADYASISGLSGQP

. . : . .

Sul.tokoda ----HTLFLVETSFNYKFRVVKKSDEGRGEVLM---------QYGNYDVTVKDLYVKGDV

M.sedula ----DQIYVLETSFDTSYTISRIKDQ-RVEVLR---------RGREERLTVKEIYVQGDV

T.barophil ----EQVLLRYESFTVPYRLYRFRDRLELIDEI---------KVEGEFEVSEDFATSKDG

T.sibiricu ----KTAILRYESFTIPYRLYKFDDNLELIDEV---------KIEWHFTVNEDFATSKDG

T.kodakare ----KKALLRYESFTVPYRLYQFDGELKLIGTQ---------EVEGKFTVEEDFATSKDG

T.onnurine ----KKVILRYESFTIPYRLYEFDGELKLLDER---------KIEGDFQVSEDFAISKDG

T.gammatol ----KKVLLRYESFTVPYRLYEFNGELKLVERQ---------EIEGSFNVEEDFAVSKDG

P.yayanosi ----GKALLRYESFTVPYRLYEFDGELKLIDEL---------KIGGNFKVEEDFAVSKDG

P.furiosus ----ERVLLRYTSFTIPYRLYEFKDDLRLIEER---------KVEGEFRVEEDFATSKDG

P.horikosh ----KEVLLRYISFTIPYRLYEFKDKLRIIEER---------KVEGKFKVEEDFVVSKDG

P.abyssi ----ERALLRYTSFTVPYRIYEFKDELKIVEER---------KVEGNFKVEEDFAISKDG

A.boonei ----G-ILLKYESFTIPSRLYEFKDSMRIIQET---------KIEGEYKIKEDFVRSKDG

Sp.thermop ESEGDRAFFRFESFTVPPTVFQCDTASGRITEWAAVEAPIDPAAYTTE---QVWYRSADG

H.paucihal SMDEDECFFRYQSFDHPPTVYRYVVGEG-VTKLDAPEVPVE-ADLQVE---RERYESADG

D.radiodur --DSSEVFVAFTSFLVPSRPYRLKLPGGELEP--LADPALD-FDAATYEVTQEFAMSKDG

D.maricope --DDPDVFLTLTSFISPPTPHHLRVPDGALEG--LADIPLA-FDPAAFEVRQDFARSRDG

Sus.scrofa --KDTEIFYQFTSFLSPGIIYHCDLTKEELEPRVFREVTVKGIDASDYQTVQIFYPSKDG

F.meningos --TEKELYYSFTNYITPPTIFKFSIDSGKSEV--YQKPKVK-FNPENYVSEQVFYTSADG

N.thermoph --NDPEMFIAYNTLLRPTTILRYTFD-GESEI--YKTPEIS-YELRDFESKQIFYESKDG

. . . . *

Sul.tokoda LLHGFLVSKAN--NP---KGVIVYGYGGFRIPLLPSLTSVMRVLLN-EGYSILITNLRGG

M.sedula LLHGFLLSKGG--N----RGVVVYGYGGFAIPLLPSYNPLFLELMD-SGYSVLVTNLRGG

T.barophil TRVHYFMVKKKGTKS---DKAWVFGYGGFNIALLPRFVPQVIPFIE-RGGTFVMTNLRGG

T.sibiricu TKIHYFIVRGE-KEE---KKAWVFGYGGFNISLKPRFFPHVIPFIE-MGGTFAMANLRGG

T.kodakare TKIHYFHVKGE-KDD---KKVWVFGYGGFNISLTPRFFPQAIPFIR-RGGTFAMANLRGG

T.onnurine TRIHYFIVKGE-KDE---KKAWVFGYGGFNIALTPRFFPQVIPFIR-RGGTFVMANLRGG

T.gammatol TKIHYFLVKGE-RDE---KKAWVFGYGGFNISLTPRFFPQAIPFIK-RGGTFGMANLRGG

P.yayanosi TRIHYFIVKGE-REE---KKAWVFGYGGFNIALTPRFFPQVIPFIR-RGGAFVMANLRGG

P.furiosus TKVHYFIVKGE-RDE---KRAWVFGYGGFNIALTPMFFPQVIPFLK-RGGTFIMANLRGG

P.horikosh TRIHYFIIKGE-KDE---KKAWVFGYGGFNIALTPRFFPQVIPFLK-RGGTFVMANLRGG

P.abyssi TRVHYFIVKGE-KDE---KKAWVFGYGGFNISLTPRFFPQVIPFLK-RGGIFVMANLRGG

A.boonei TNIHYFLVEGK-NKN---NIAWVFGYGGFNISLSPRFFPHVVPFLK-RGGTFVVANLRGG

Sp.thermop TLVSMFIVARAGTPRDGSAPALLTGYGGFNISRTPLFDRRMFFWLE-QGGVYALPNLRGG

H.paucihal TEVPLFVVH-AGVEPDGDNPTLLYGYGGFELSQTPAFRRYAVPFLE-RGGVFVVANVRGG

D.radiodur TRVPMFIVARKDAPRDGSNRTLLYGYGGFSISLTPAFSASRLAWLE-RGGVFVQANLRGG

D.maricope TRVPYFTVRRRDLTPGTPHPTLLYGYGGFNHSLLPSFSLQTVAWVE-RGGVYVSANLRGG

Sus.scrofa TKIPMFIVHKKGIKLDGSHPAFLYGYGGFNISITPNYSVSRLIFVRHMGGVLAVANIRGG

F.meningos TKIPMMISYKKGLKKDGKNPTILYSYGGFNISLQPAFSVVNAIWME-NGGIYAVPNIRGG

N.thermoph TQVPMFLIYKKGLELNGNNPALIFGYGGFKISMNPRFSPANIKWIE-EGGIFAIACIRGG

: . : .**** . * : * . :***

Sul.tokoda YENGEEWHKAGMLLNKKNVFKDFAEFLRLV----KLMGGKAVAMGGSNGGLLVGATINEY

M.sedula FENGEEWHKAGMLRNKMNVFKDFSEFLQTV----KMMGGRTIAMGGSNGGLLVGATLNLY

T.barophil SEYGEEWHRQGMRENKQNVFDDFIAVLEKL----KREGYKVAAWGRSNGGLLVSAVLVQR

T.sibiricu SEYGEEWHKAGMRENKQNVFDDFIAVLEKL----KEEDYKVAAWGRSNGGLLVSTVLVQR

T.kodakare SEYGEEWHRAGMRENKQNVFDDFIAVLEKL----KKEGYRIAAWGRSNGGLLVSATLTQR

T.onnurine SEYGEEWHRAGMRENKQNVFDDFIAVLGKL----KAEGYKVAAWGRSNGGLLVSATLVQR

T.gammatol SEYGEEWHRAGMRENKQNVFDDFIAVLEKL----KREGYRVAAWGRSNGGLLVSATLVQR

P.yayanosi SEYGEEWHRAGMRENKQNVFDDFIAVLEKL----KGKGYRVAAWGRSNGGLLVSATLVQR

P.furiosus SEYGEEWHRAGMRENKQNVFDDFIAVLEKR----KKEGYKVAAWGRSNGGLLVSATLTQR

P.horikosh SEYGEEWHRAGMRENKQNVFDDFIAVLEKL----KKEGYKVAAWGRSNGGLLVSATLTQR

P.abyssi SEYGEEWHRAGMRENKQNVFDDFIAVLEKL----KKEGYKVAAWGRSNGGLLVSATLVQR

A.boonei SEYGEEWHRAGMRDKKQNVFDDFISVLENL----KKD-YKVVAWGRSNGGLLVAATLVQR

Sp.thermop GEYGEEWHRAGMRERKQNVFDDFIAAAEYLIAEGYTRPERLAILGGSNGGLLVGAAMTQR

H.paucihal GEFGEEWHHAARKEHKQNTFDDFIAAAEHLVESDYTSPERLAIDGRSNGGLTVGASITQR

D.radiodur GEYGEAWHEAGTLGRKQNVFDDFAACAEHLARQGWTRPERLAIQGGSNGGLLVGASITQR

D.maricope SEYGRAWHQAGTRERKQNVFNDFIAVAEDLIERGVTTPARLAINGGSNGGLLVGACLTQR

Sus.scrofa GEYGETWHKGGILANKQNCFDDFQCAAEYLIKEGYTSPKRLTINGGSNGGLLVATCANQR

F.meningos GEYGKKWHDAGTKQQKKNVFNDFIAAGEYLQKNGYTSKDYMALSGRSNGGLLVGATMTMR

N.thermoph NEYGEDWHRQGMLLNKQNVFDDFIAAGEWLIDNNYTRKDKLAITGRSNGGLLVAACMTQR

* *. ** . .* * *.** * ***** *.:

Sul.tokoda PELIDCAVIGHPVLDMLRYDKLYVGKYWVEEYGDP-NDPKYTEYLLSYSPYHNLKK----

M.sedula TSLVDCGVIGYPVLDMLKFHKYLAGMYWVPEYGDP-E--KDSEFLLSYSPYHNLKK----

T.barophil PDVMDAALIGYPVIDMLRFHKLYIGSVWIPEYGNP-DEPKDRKFLLKYSPYHNVKP----

T.sibiricu PDVMDAALIGYPVIDMLRFHKLYIGSVWVPEYGNP-DDPRDREFLLKYSPYHNIDPT---

T.kodakare PDVMDAALIGYPVIDMLRFHKLYIGSVWIPEYGNP-DDPKDREFLLKYSPYHNVDPN---

T.onnurine PDVMDAALIGYPVIDMFRFHKLYIGSVWVPEYGNP-DDPKDREFLLKYSPYHNVKEQ---

T.gammatol PDVMDSALIGYPVIDMLRFHRLYIGSVWVPEYGNP-DDPKEREFLLKYSPYHNVKPA---

P.yayanosi PEVMDSALIGYPVIDMMRFHKLYIGSVWIPEYGNP-DDPKDREFLLKYSPYHNVKPQ---

P.furiosus PDVMDSALIGYPVIDMLRFHKLYIGSVWIPEYGNP-EDPKDREFLLKYSPYHNVDPK---

P.horikosh PDIMDAALIGYPVIDMLRFHKLYIGSVWIPEYGNP-DDPKDREFLLKYSPYHNVDPN---

P.abyssi PDVMDVALIGYPVIDMLRFHKLYIGSVWIPEYGNP-DDPKDREFLLKYSPYHNVRP----

A.boonei PDVMDAAIIGYPVIDMLRFHRMYIGRVWIPEYGNP-DEPEDRKFLEKYSPYHNVKK----

Sp.thermop PDLFRAVVCQVPLLDMLRYHRFLIARLWIPEYGSA-DDPEQFAYLYAYSPYHRVEDG---

H.paucihal PDLFAACLCIVPLLDMLRFHRFLLGASWTAEYGSP-DDPEAFEYIREYSPYHNVEE----

D.radiodur PELFGAAVAQVGVLDMLRYHLFTIGWAWASDYGRS-DDPEMFATLHAYSPLHNLKE----

D.maricope PDLFGAAIPMVGVLDMLRYHQFTIGWAWASDYGTS-DDPDGYRTLRRYSPLHNVQP----

Sus.scrofa PDLFGCVIAQVGVMDMLKFHKYTIGHAWTTDYGCS-DSKQHFEWLIKYSPLHNVKLPEAD

F.meningos PDLAKVAFPGVGVLDMLRYNKFTAGAGWAYDYGTAEDSKEMFEYLKSYSPVHNVKA----

N.thermoph PDLYGAVVCGVPVIDMLRFHKFTIGRYWIPEYGDPDNDPQAFENLYSYSPLHNISK----

..: . ::**:::. . * :** . : : *** *.:

Sul.tokoda --GLPKTFVYTGINDDRVHPAHALKYVAKSKSLGND-------VMLFVND-SGHSIADP-

M.sedula --GLPPTLVYTGLNDDRVHPMHALKYVAKSREMGNK-------VYLFVNRRAGHNLSRP-

T.barophil -QKYPPTLIYTGLHDDRVHPAHALKFAKKLKDVGAP-------VYLRVETKSGHMGASP-

T.sibiricu -KKYPPTLIYTGLHDDRVHPGHALKFAKRLRDMGAP-------VYLRVETKSGHMGASP-

T.kodakare -KRYPPTLIYTGLHDDRVHPAHALKFFMKLREVGAP-------VYLRVETKSGHMGASP-

T.onnurine -K-YPPTLLYTGLYDDRVHPAHALKFFMKLRAVSAP-------VYLRVETKSGHMGASP-

T.gammatol -K-YPPTLIYTGLHDDRVHPAHAIKFFLKLKEVSRD-------VYLRVEIKSGHMGASP-

P.yayanosi -K-YPPTLIYTGLYDDRVHPAHALKFFMRLKEVGAP-------VYLRVETKSGHMGASP-

P.furiosus -KKYPPTLIYTGLHDDRVHPAHALKFFMKLKEIGAP-------VYLRVETKSGHMGASP-

P.horikosh -KKYPLTLIYTGLHDDRVHPAHALKFFMKLKEVGAP-------VYLRVETKSGHMGASP-

P.abyssi -QEYPPTLIYTGLHDDRVHPAHALKFFMKLKEVNAP-------VYLRVETKSGHMGASP-

A.boonei -KNYPPIMIYTGLHDDRVHPAHALKFFMKLREVDAP-------VYLRVETKSGHMGASP-

Sp.thermop -TPYPAVLLTTATSDTRVAPLHARKMAARLQAATGS----DLPVLLRVETAAGHGAGKPL

H.paucihal -REYPAVLFKTAEGDTRVHPAHARKMAARMQAKHTG----DAPILLREERDTGHGVGKPT

D.radiodur GTRYPATLITTGDHDDRVVPAHSYKFAAELQRVQAG----SAPTLIRIQTRAGHGAGKPT

D.maricope GTAYPPTLITTGDHDDRVVPAHSYKFAAALQHAQAG----HAPILLRVGRQAGHGAGKPT

Sus.scrofa DIQYPSMLLLTADHDDRVVPLHSLKFIATLQYIVGRSRKQNNPLLIHVDTKAGHGAGKPT

F.meningos GTCYPSTMVITSDHDDRVVPAHSFKFGAELQAKQAC----KNPVLIRIETNAGHGAGRST

N.thermoph GEVYPHTLVLTADTDDRVVPAHALKFVRALKDNAKN----NQDIFLRMEKKAGHGLGKPI

* :. *. * ** * *: * : : :** . .

Sul.tokoda ESKAREESYVVSFIEECLR-----

M.sedula EASAEEMSTVVAFVEQCHSL----

T.barophil ETRIKELADLLAFVIKVLGVD---

T.sibiricu ETRIRELADMLSFVVKTLEVKV--

T.kodakare ETRARELTDLLAFVVKTLR-----

T.onnurine ETRARELTDLLAFVVETLEA----

T.gammatol ETRARELTDLLAFVLRTLA-----

P.yayanosi ETRAKELADLLAFVVKTLGV----

P.furiosus ETRARELTDLLAFVLKTLS-----

P.horikosh ETRARELTDLLAFVLKSLS-----

P.abyssi ETRARELTDLLAFVLLHL------

A.boonei ETRIRELADILAFVLKSTGVLN--

Sp.thermop GKQIAEQTDIWTFVCDQLGVTVA-

H.paucihal EMIVREKLDEWTFVFEQLGVE---

D.radiodur ALVIEEAADIWAFLEEVLGG----

D.maricope HLKIEEQADILAFLAATVGQNHTP

Sus.scrofa AKVIEEVSDMFAFIARCLNIDWIP

F.meningos EQVVMENADLLSFALYEMGIKNLK

N.thermoph GKRIEEDADWLSFLLKVL------

* :*

**Figure S3**. Phylogenetic tree of prolyl endopeptidases. (http://www.phylogeny.fr/). Seventeen sequences from thermophilic organisms were analyzed along with sequences of *Flavobacterium meningosepticum* and *Sus scrofa* prolyl endopeptidases. The latter sequence was used as a reference for positioning relevant amino acid residues.


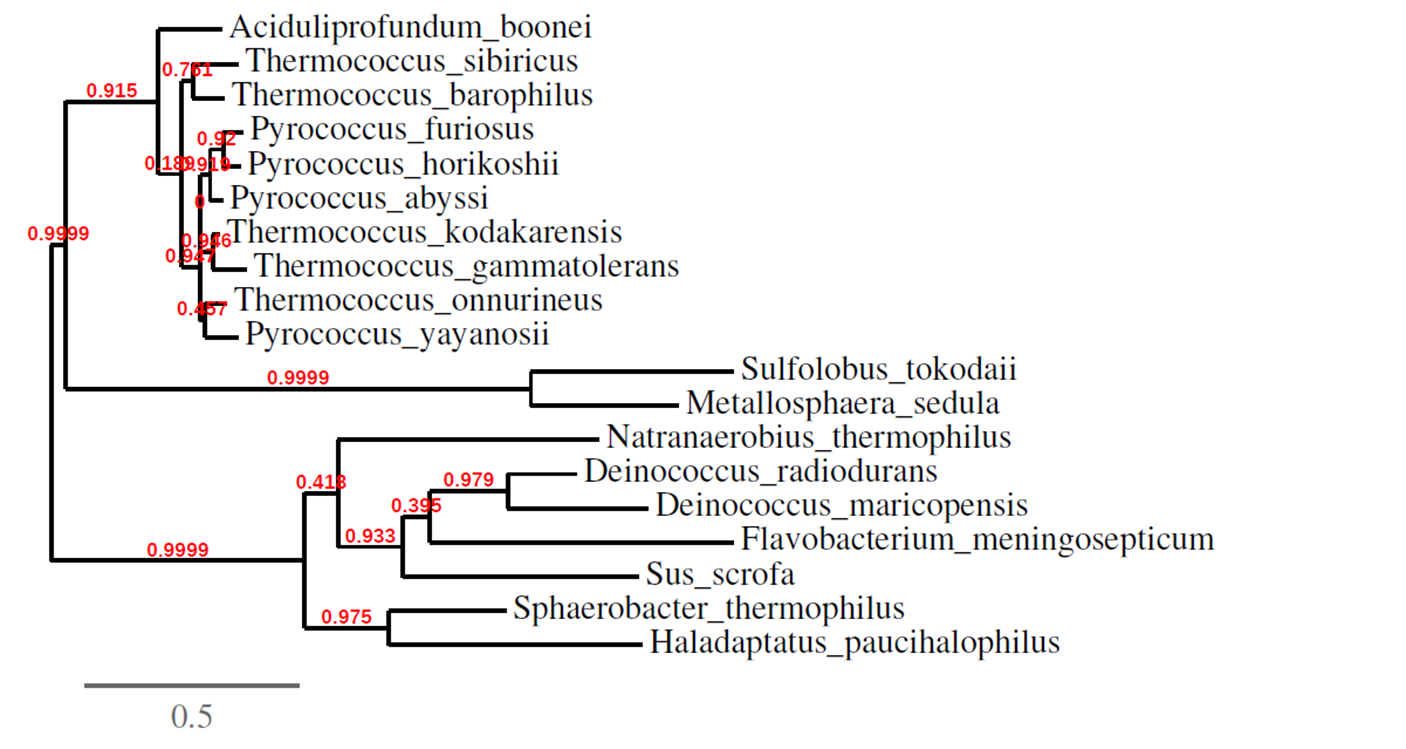


**Figure S4.** Combination of mutations involving amino acid residues at 412-415. The Fm-PEP variants carrying these mutations showed stability at higher temperatures likely due to the formation of the hydrogen bond, as witnessed in the in-silico structural analysis of mutant enzymes. Yellow arrows show newly formed hydrogen bonds (A) at amino acid residues 412 and 413 and (B) at amino acid residue 415.

A B

**Figure S5.** Effect of temperature on EP-B2 stability. Enzyme activity was measured under different temperature treatments using a synthetic substrate. Absorbance was recorded in triplicate, and averages are shown in the graphs.


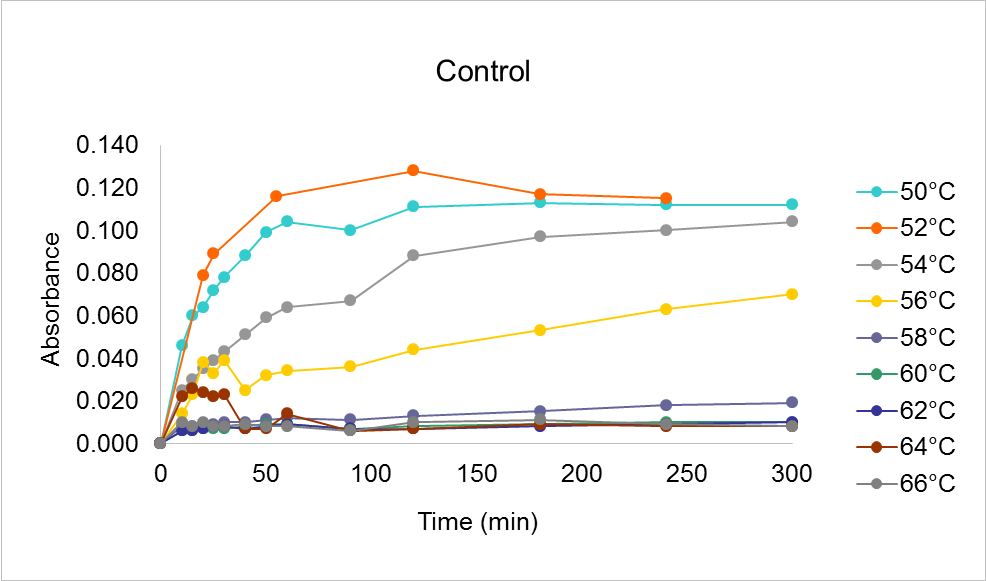

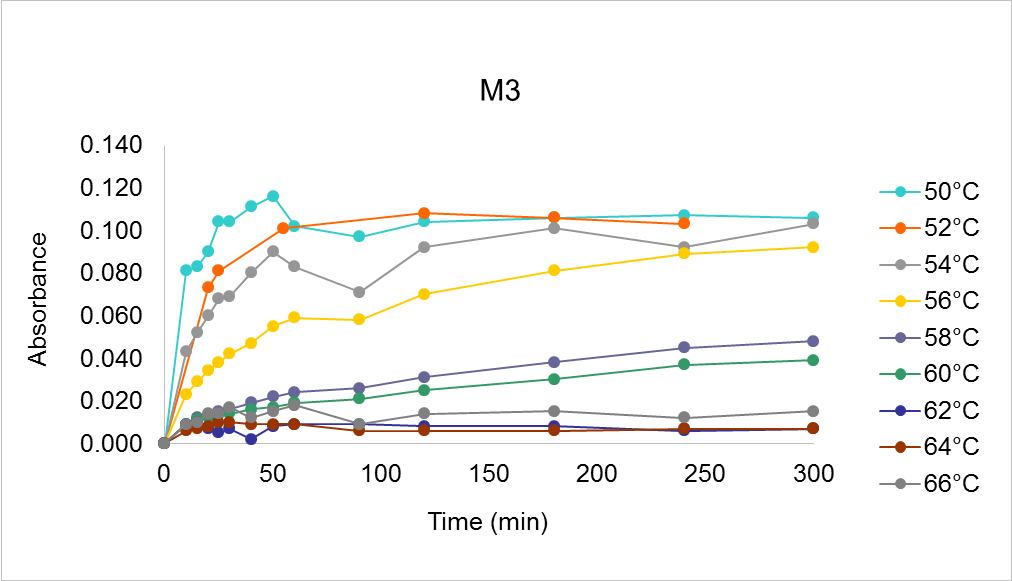


**Table S1.** List of primers used to introduce mutations in the *Flavobacterium meningosepticum* prolyl endopeptidase and *Hordeum vulgare* cysteine endopeptidase (EP-B2) gene sequences.

| **Primer** | **Sequence 5' to 3'** |
| --- | --- |
| *Nde*I | ATCCCACTGAACTTGACGTCGCTGCCGCGCGGCACCA |
| *Aat*II-1 | CGCGCGGCAGCCATATGAAGTTCAGTGGGATCTC |
| F123-125 | CGTGCTCTACATCAAGTGGGCGGCGG |
| R123-125 | CCGCCGCCCACTTGATGTAGAGCACG |
| F136-137 | GTTCTTGGACGAGCTCAAGTTCTCGG |
| R136- 137 | CCGAGAACTTGAGCTCGTCCAAGAAC |
| F406 | CCTTCACCAGCTACATCACGCCGCCG |
| R406 | CGGCGGCGTGATGTAGCTGGTGAAGG |
| F412-413 | GCCGCCGAGGCTCTTCAAGTACAAC |
| R412-413 | GTTGTACTTGAAGAGCCTCGGCGGC |
| F412/415 | GCCGCCGAGGCTCTACGAGTACAAC |
| R412/415 | GTTGTACTCGTAGAGCCTCGGCGGC |
| F414-415 | GCCGCCGACGATCTACGAGTACAAC |
| R414-415 | GTTGTACTCGTAGATCGTCGGCGGC |
| *Aat*II-2 | GTACTCCCCGCCGCCGCGGAAGTTCAGTGGGATCTC |
| *Sac*II | ACGAGACCCTGTTGGACGTCATGTTCGGGACGGCGTA |
| F1 | GCGGCCGTGGATCCCTGCAGCG |
| R1 | CGATCGATCAGTGAATTCAGTGACTCCCTGGCTCC |
| FM1&2 | TGTCGTCCGTGGAAAGCATCAAC |
| RM1&2 | ATGCTTTCCACGGACGACACCGTG |
| FM3 | GCGCGGCGTACTGGACGGTGAAGAACT |
| RM3 | TACGCCGCGCCGTCCTCTG |

**Table S2.** List of prolyl endopeptidase gene accession numbers derived from thermophilic organisms, used as reference in the present study.

| **Organism** | **Optimal growth (°C)** | **Accession number** |
| --- | --- | --- |
| *Thermococcus barophilus* | 48- 95 | YP_004070728 |
| *Thermococcus sibiricus* | 60- 84 | YP_002993586 |
| *Pyrococcus furiosus* | 100 | AAA73423.1 |
| *Pyrococcus horikoshii* | 98 | NP_143154.1 |
| *Pyrococcus abyssi* | 102 | NP_126828.1 |
| *Thermococcus kodakarensis* | 86 | YP_182836.1 |
| *Thermococcus onnurineus* | 80-90 | YP_002306995 |
| *Thermococcus gammatolerans* | 88 | YP_002959404 |
| *Pyrococcus yayanosii* | 98 | AEH24451.1 |
| *Aciduliprofundum boonei* | 70 | ZP_04875685.1 |
| *Sulfolobus tokodaii* | 80 | NP_375840.1 |
| *Metallosphaera sedula* | 75 | YP_001191382 |
| *Sphaerobacter thermophiles* | 65 | YP_003320677 |
| *Haladaptatus paucihalophilus* | 45 | ZP_08043415.1 |
| *Deinococcus maricopensis* | 45 | YP_004171970 |
| *Natranaerobius thermophiles* | 57 | YP_001918125 |

**Table S3.** List of catalytic residues conserved among *Flavobacterium meningosepticum* prolyl endopeptidases (Fm-PEP) and thermophilic organisms used as reference in the present study. Level of similarity is shown as pertentage.

| **Location** | **Position** | **Function** | **Fm-PEP** | **Thermophilic organisms** | **% Similarity** |
| --- | --- | --- | --- | --- | --- |
| **S1** | 527 | Catalysis | Ser | Ser | 100 |
|  | 528 | H- bond | Asn | Asn | 100 |
|  | 611 | Catalysis | Asp | Asp | 100 |
|  | 675 | Catalysis | His | His | 100 |
|  | 568 | Ring stacking | Trp | Trp | 100 |
|  | 476 | H- bond | Tyr | Tyr | 100 |
|  | 479 | Lining | Phe | Phe | 100 |
|  | 643 | Lining | Val | Val | 100 |
|  | 584 | Lining | Val | Val | 88.9 |
|  | 603 | Lining | Tyr | Tyr | 100 |
| **S2** | 642 | H- bond | Arg | Arg | 100 |
| **S3** | 568 | H- bond | Trp | Trp | 100 |
|  | 193 | Lining | Phe | Phe | 27.8 |
|  | 252 | Lining | Tyr | Phe | 61.11 |
|  | 272 | Lining | Thr | Trp | 77.8 |
|  | 594 | Lining | Ala | Ile | 83 |
|  | 597 | Lining | Gly | Val | 55.5 |

**Table S4.** Location of amino acid substitutions in Fm-PEP sequence and activity of Fm-PEP variants, measured using synthetic substrate, Z-Gly-Pro-pNA after heat shock for 10 minutes at 80ºC. For activity assays, absorbance at 410 nm was recorded. The Beer-Lambert equation (A= ε c l) and the concentration of the product was calculated based on the extinction coefficient for pNa (8.8mM).

| **Variant** | **Residue** | | | | | | | | | **Activity**  **(μM product)** | **Stdv** |
| --- | --- | --- | --- | --- | --- | --- | --- | --- | --- | --- | --- |
|  | **123** | **125** | **136** | **137** | **406** | **412** | **413** | **414** | **415** |  |  |
| WT |  |  |  |  |  |  |  |  |  | 1.5417 | 0.0741 |
| Fme2 | X | X |  |  |  |  |  |  |  | 0.8950 | 0.0060 |
| Fme3 |  |  | X | X |  |  |  |  |  | 3.1497 | 0.0057 |
| Fme4 |  |  |  |  | X |  |  |  |  | 0.2280 | 0.0030 |
| Fme5 |  |  |  |  |  | X | X |  |  | 5.8420 | 0.0892 |
| Fme6 |  |  |  |  |  |  |  | X | X | 4.2357 | 0.0977 |
| Fme7 |  |  |  |  |  | X | X | X | X | 2.5617 | 0.0921 |
| Fme8 | X | X | X | X |  |  |  |  |  | 2.2637 | 0.0336 |
| Fme9 |  |  |  |  | X | X | X |  |  | 2.5950 | 0.0275 |
| Fme10 |  |  |  |  | X | X | X | X | X | 3.7717 | 0.0994 |
| Fme11 | X | X |  |  | X |  |  |  |  | 0.1053 | 0.0055 |
| Fme12 |  |  | X | X | X |  |  |  |  | 0.0561 | 0.0051 |
| Fme13 | X | X | X | X | X |  |  |  |  | 0.0711 | 0.0107 |
| Fme14 | X | X |  |  | X | X | X |  |  | 1.3657 | 0.0581 |
| Fme15 | X | X |  |  | X |  |  | X | X | 0.6087 | 0.0100 |
| Fme16 | X | X |  |  | X |  |  | X | X | 3.6067 | 0.3399 |
| Fme17 | X | X |  |  | X | X | X | X | X | 0.0117 | 0.0015 |
| Fme18 |  |  | X | X | X | X | X |  |  | 4.2033 | 0.0208 |
| Fme19 |  |  | X | X | X |  |  | X | X | 0.2683 | 0.0131 |
| Fme20 |  |  | X | X | X | X | X | X | X | 0.5077 | 0.0061 |
| Fme21 | X | X |  |  | X | X | X | X | X | 0.8473 | 0.0412 |
| Fme22 | X | X | X | X | X |  |  |  |  | 0.6600 | 0.0060 |
| Fme23 | X | X | X | X |  | X | X |  |  | 1.6743 | 1.1694 |
| Fme24 | X | X | X | X |  |  |  | X | X | 0.3327 | 0.0186 |
| Fme25 | X | X | X | X |  | X | X | X | X | 1.1140 | 0.0010 |
| Fme26 | X |  |  |  | X |  |  |  |  | 0.4080 | 0.0020 |
| Fme27 | X |  |  |  |  | X |  |  |  | 0.1493 | 0.0059 |
| Fme28 | X |  |  |  |  |  | X |  |  | 0.1500 | 0.0056 |
| Fme29 | X |  |  |  |  |  |  | X |  | 0.0943 | 0.0040 |
| Fme30 | X |  |  |  |  |  |  |  | X | 0.0850 | 0.0053 |
| Fme31 |  | X |  |  | X |  |  |  |  | 0.0589 | 0.0012 |
| Fme32 |  | X |  |  |  | X |  |  |  | 0.0763 | 0.0023 |
| Fme33 |  | X |  |  |  |  | X |  |  | 0.0150 | 0.0020 |
| Fme34 |  | X |  |  |  |  |  | X |  | 0.0231 | 0.0009 |
| Fme35 |  | X |  |  |  |  |  |  | X | 0.0332 | 0.0011 |
| Fme36 |  |  | X |  | X |  |  |  |  | 0.0379 | 0.0010 |
| Fme37 |  |  | X |  |  | X |  |  |  | 0.0289 | 0.0001 |
| Fme38 |  |  | X |  |  |  | X |  |  | 0.0186 | 0.0006 |
| Fme39 |  |  | X |  |  |  |  | X |  | 0.0169 | 0.0002 |
| Fme40 |  |  | X |  |  |  |  |  | X | 0.2757 | 0.0059 |
| Fme41 |  |  |  | X | X |  |  |  |  | 0.4913 | 0.0032 |
| Fme42 |  |  |  | X |  | X |  |  |  | 1.0947 | 0.0055 |
| Fme43 |  |  |  | X |  |  | X |  |  | 0.3147 | 0.0025 |
| Fme44 |  |  |  | X |  |  |  | X |  | 0.2257 | 0.0032 |
| Fme45 |  |  |  | X |  |  |  |  | X | 0.4327 | 0.0103 |
| Fme46 | X |  | X |  |  |  |  |  |  | 0.3860 | 0.0061 |
| Fme47 | X |  |  | X |  |  |  |  |  | 0.2147 | 0.0127 |
| Fme48 |  | X | X |  |  |  |  |  |  | 0.1473 | 0.0021 |
| Fme49 |  | X |  | X |  |  |  |  |  | 0.9897 | 0.0083 |
| Fme50 | X |  | X |  | X |  |  |  |  | 0.8043 | 0.0045 |
| Fme51 | X |  |  | X | X |  |  |  |  | 0.1183 | 0.0021 |
| Fme52 |  | X | X |  | X |  |  |  |  | 0.0923 | 0.0049 |
| Fme53 |  | X |  | X | X |  |  |  |  | 0.0743 | 0.0047 |
| Fme54 | X |  | X |  | X | X | X |  |  | 0.0675 | 0.0012 |
| Fme55 | X |  |  | X | X | X | X |  |  | 0.0586 | 0.0017 |
| Fme56 |  | X | X |  | X | X | X |  |  | 0.0440 | 0.0014 |
| Fme57 |  | X |  | X | X | X | X |  |  | 0.3537 | 0.0078 |
| Fme58 | X |  | X |  | X |  |  | X | X | 0.3173 | 0.0042 |
| Fme59 | X |  |  | X | X |  |  | X | X | 0.0153 | 0.0031 |
| Fme60 |  | X | X |  | X |  |  | X | X | 0.0017 | 0.0012 |

**
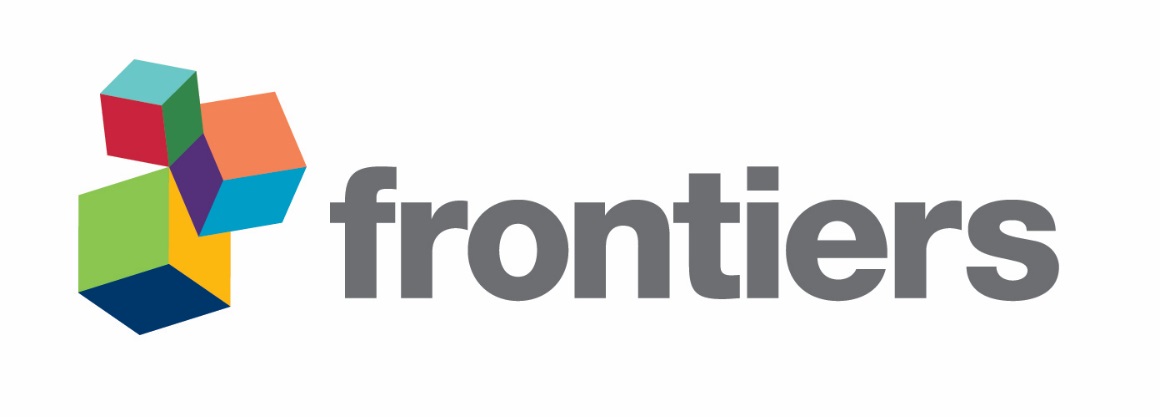
**
